# Current Implementation of Digital Health in Chronic Disease Management: Scoping Review

**DOI:** 10.2196/53576

**Published:** 2024-12-12

**Authors:** Candelyn Pong, Rachel Marjorie Wei Wen Tseng, Yih Chung Tham, Elaine Lum

**Affiliations:** 1 Health Services and Systems Research Duke-NUS Medical School National University of Singapore Singapore Singapore; 2 Singapore Eye Research Institute Singapore National Eye Centre Singapore Singapore; 3 Duke-NUS Medical School National University of Singapore Singapore Singapore; 4 Centre for Innovation and Precision Eye Health Yong Loo Lin School of Medicine National University of Singapore Singapore Singapore; 5 Department of Ophthalmology Yong Loo Lin School of Medicine National University of Singapore Singapore Singapore; 6 Centre for Population Health Research and Implementation SingHealth Singapore Singapore

**Keywords:** digital health, telemedicine, chronic disease, noncommunicable disease, implementation science, evidence-based practice, mobile phone

## Abstract

**Background:**

Approximately 1 in 3 adults live with multiple chronic diseases. Digital health is being harnessed to improve continuity of care and management of chronic diseases. However, meaningful uptake of digital health for chronic disease management remains low. It is unclear how these innovations have been implemented and evaluated.

**Objective:**

This scoping review aims to identify how digital health innovations for chronic disease management have been implemented and evaluated: what implementation frameworks, methods, and strategies were used; how successful these strategies were; key barriers and enablers to implementation; and lessons learned and recommendations shared by study authors.

**Methods:**

We used the Joanna Briggs Institute methodology for scoping reviews. Five databases were searched for studies published between January 2015 and March 2023: PubMed, Scopus, CINAHL, PsycINFO, and IEEE Xplore. We included primary studies of any study design with any type of digital health innovations for chronic diseases that benefit patients, caregivers, or health care professionals. We extracted study characteristics; type of digital health innovation; implementation frameworks, strategies, and outcome measures used; barriers and enablers to implementation; lessons learned; and recommendations reported by study authors. We used established taxonomies to synthesize extracted data. Extracted barriers and enablers were grouped into categories for reporting. Descriptive statistics were used to consolidate extracted data.

**Results:**

A total of 252 studies were included, comprising mainly mobile health (107/252, 42.5%), eHealth (61/252, 24.2%), and telehealth (97/252, 38.5%), with some studies involving more than 1 innovation. Only 23 studies (23/252, 9.1%) reported using an implementation science theory, model, or framework; the most common were implementation theories, classic theories, and determinant frameworks, with 7 studies each. Of 252 studies, 144 (57.1%) used 2 to 5 implementation strategies. Frequently used strategies were “obtain and use patient or consumer feedback” (196/252, 77.8%); “audit and provide feedback” (106/252, 42.1%); and piloting before implementation or “stage implementation scale-up” (85/252, 33.7%). Commonly measured implementation outcomes were acceptability, feasibility, and adoption of the digital innovation. Of 252 studies, 247 studies (98%) did not measure service outcomes, while patient health outcomes were measured in 89 studies (35.3%). The main method used to assess outcomes was surveys (173/252, 68.7%), followed by interviews (95/252, 37.7%). Key barriers impacting implementation were data privacy concerns and patient preference for in-person consultations. Key enablers were training for health care workers and personalization of digital health features to patient needs.

**Conclusions:**

This review generated a summary of how digital health in chronic disease management is currently implemented and evaluated and serves as a useful resource for clinicians, researchers, health system managers, and policy makers planning real-world implementation. Future studies should investigate whether using implementation science frameworks, including how well they are used, would yield better outcomes compared to not using them.

## Introduction

### Background

Chronic diseases are among the biggest health threats, causing approximately 41 million deaths yearly [1]. Chronic disease management is especially difficult because these diseases do not occur in isolation; approximately 1 in 3 adults have multiple chronic conditions [2], and the probability of developing multiple comorbidities increases with age [3].

Digital health is a rapidly growing industry that could potentially enhance health outcomes, with its growth accelerated by the COVID-19 pandemic. The use of digital health enabled continuity of care during the COVID-19 pandemic, especially for chronic disease management, when in-person care was limited [4,5]. An estimated 20% or US $1.8 trillion of the world’s health care spending is wasteful, and digital health has the potential to lower health care spending by tackling this waste [6]. Despite its benefits, meaningful uptake of digital health for chronic disease management has been relatively low [7]. Some known barriers to adoption include lack of access and availability of digital health [8], poor user interface, suboptimal clinical integration, and lack of transparency about the datasets used for the development of digital health tools [9].

The uptake of health care innovations can be addressed using implementation science. Implementation science is defined as the scientific investigation of ways to increase the adoption of evidence-based practices and research findings into routine clinical use [10]. It is underpinned by theoretical frameworks collectively known as implementation science theories, models, and frameworks (henceforth, for simplicity, implementation science frameworks). Nilsen’s taxonomy of implementation science frameworks shows 5 categories (Textbox 1): process models, determinant frameworks, classic theories, implementation theories, and evaluation frameworks [11].

**Descriptions and examples of theories, models, and frameworks used in implementation science.**

- Process models: These models are used to detail and facilitate the process of bridging the gap between research and routine clinical practice. Examples are the Knowledge To Action framework [12] and the Ottawa Model [13].
- Determinant frameworks: These are frameworks that provide a list of factors for analysis that could affect implementation outcomes. Examples are Theoretical Domains Framework [14] and Consolidated Framework for Implementation Research [15].
- Classic theories: These are theories used to analyze the factors that shape implementation outcomes, usually drawn from other disciplines, such as psychology and organizational theory [11]. Examples are the Theory of Planned Behavior [16], Social Cognitive Theory [17], and Situated Change Theory [18].
- Implementation theories: These are theories generated from implementation research to understand and address elements that shape implementation outcomes. An example is Normalization Process Theory [19].
- Evaluation frameworks: These are frameworks to guide the evaluation of implementation efforts. Examples are the RE-AIM (reach, effectiveness, adoption, implementation, and maintenance) framework [20] and the framework for Outcomes in Implementation Research [21].

Prospective use of implementation science frameworks can aid the development, implementation, and evaluation of digital health innovations [11]. Retrospective application of implementation science frameworks, although less common, can be used to understand an innovation’s success or failure [22]. Given the plethora of implementation science frameworks available, the choice of framework largely depends on the study aim. For example, if the study aims to prospectively explore potential barriers and enablers to the implementation of an innovation, a determinant framework, such as the Consolidated Framework for Implementation Research, would be appropriate. The advantage of underpinning real-world implementation efforts with an appropriate implementation science framework is the ability to generate reliable translation, spread, and scale-up for evidence-based innovations [23].

### Objectives

Despite the usefulness of implementation science, there is a scarcity of reviews on the implementation [24] and evaluation of digital health innovations for chronic disease management in real-world or clinical settings. Most reviews are relatively narrow, focusing on a specific element of implementation such as barriers, a specific chronic disease, or a specific type of digital health innovation [25-28]. In light of the current knowledge gaps, this scoping review aimed to identify how digital health innovations for the management of chronic diseases have been implemented. Specifically, to understand what implementation frameworks, methods, and strategies were used; how successful these strategies were; what were the key barriers and enablers to implementation; what lessons were learned; and what recommendations were shared by the respective study authors. Findings will be useful to clinicians and researchers planning to implement digital health innovations.

## Methods

### Definitions

For the purposes of this scoping review, we defined digital health as the branch of study on the advancement and use of IT to enhance health [29]. This includes eHealth, mobile health (mHealth), telehealth, wearables, and artificial intelligence.

eHealth refers to the provision of health care services with the support of information and communication technology (ICT), for example, computers and phones; mHealth is defined as the use of smart and portable or mobile wireless devices in health care; and telehealth is defined as the use of digital technologies to provide health services remotely [29,30]. Wearables are devices worn by individuals, usually to monitor personal health metrics and their environment [31]. Artificial intelligence in health care refers to the use of machine learning, including natural language processing and deep learning, to make predictions of health outcomes or support clinical decision-making [32].

### Search Strategy

This study was carried out according to the Joanna Briggs Institute methodology for scoping reviews [33]. The protocol was published on Open Science Framework [34,35] and briefly detailed in this paper. We developed a search strategy comprising the following key concepts: digital health, implementation, and chronic diseases, and refined it using the PRESS (Peer Review of Electronic Search Strategies) guidelines (Table S1 in Multimedia Appendix 1 [36-198]) [199]. The full search strategy is provided in Multimedia Appendix 1.

We searched the following 5 databases, PubMed, Scopus, CINAHL, PsycINFO, and IEEE Xplore, for literature published between January 2015 and March 2023. We chose to start from 2015 as there was a steep escalation in the number of published studies on digital health innovations from 2016 onward [200].

### Eligibility Criteria

We included primary studies of any study design reporting on the preimplementation or implementation of any type of digital health innovations for chronic diseases that benefits either patients, caregivers, or health care professionals directly, with or without the use of an implementation science framework.

Studies that did not include implementation or were not reported in the English language, meta-analyses, systematic reviews, conference proceedings, short reports, study protocols, commentaries, and dissertations were excluded from this review.

### Selection of Studies

Three researchers (CP, RMWWT, and EL) conducted title and abstract screening followed by full-text screening, with each study independently screened by 2 researchers. Conflicts at both stages of screening were resolved through discussion, and any unresolved conflicts were mediated by a third researcher. The reference lists of studies that partially met the inclusion criteria but were excluded because these were meta-analyses, systematic reviews, or study protocols were examined by 1 researcher (CP) to identify any additional relevant studies. Covidence (Veritas Health Innovation), a web-based collaboration platform for reviews, and Endnote (version 20; Clarivate Analytics) were used for screening and managing citations, respectively.

### Data Extraction and Data Analysis

A standardized form was developed for data extraction using Google Forms (Google LLC). The following data were extracted: publication year, author, country of study, type of study, characteristics of the digital health innovations, definitions of digital health used by study authors, implementation frameworks used, implementation strategies, and outcome measures used to evaluate the implementation. We also extracted the key barriers and enablers for successful implementation, lessons learned, and recommendations shared by the respective study authors. How we operationalized data extraction is presented in Multimedia Appendix 1. For example, textual data points, such as “key barriers,” were summarized as “patients’ lack of motivation and time,” “increased workload for health care workers,” and so forth.

The extraction form was piloted by 2 researchers (CP and RMWWT) using 5 included studies and subsequently refined. One reviewer (CP) completed data extraction for the remaining studies. Data extraction of a random 10% (25/252, 9.9%) of included articles was verified by a second researcher (EL) to ensure rigor and trustworthiness. Descriptive statistics were used to consolidate the extracted data in Excel (version 1808; Microsoft Corporation).

We used the following taxonomies to inform our analysis and summary of extracted data: the categorization for countries and regions by the World Health Organization for the country of study [201]; Nilsen’s taxonomy for implementation theories, models, and frameworks [11]; Expert Recommendations for Implementing Change (ERIC) taxonomy for implementation strategies [202]; and Proctor’s outcomes in implementation research [21]. Logic models and pathways were not considered an implementation science framework in this review.

### A Note About Outcomes

#### Overview

Outcomes were grouped into 3 categories, namely, implementation outcomes, service outcomes, and patient outcomes, following Proctor et al [21]. Implementation outcomes indicate the success (or otherwise) of implementing or embedding the digital health innovation. Service outcomes and patient outcomes indicate the effectiveness of digital health innovation in impacting service delivery or patient care and patient health or well-being, respectively. These 3 categories of outcomes are detailed in the *Implementation Outcomes* and *Service Outcomes and Patient Outcomes* sections.

#### Implementation Outcomes

Proctor’s outcomes for implementation research comprise 8 types of implementation outcomes, namely, acceptability, adoption, appropriateness, cost, feasibility, fidelity, penetration, and sustainability [21]. Acceptability is the impression among stakeholders that the specific innovation is agreeable, adoption is the initial desire to test or use a given innovation, and appropriateness is the perceived suitability of the innovation in a particular context. Cost refers to both the cost of the innovation and the cost of implementation. Feasibility is the extent to which an innovation can be effectively implemented in a specific context, and fidelity is the extent to which the innovation was implemented as intended. Penetration is the integration of the innovation into health care services, and sustainability is the degree to which an innovation and its ensuing benefits can be effectively maintained in a specific context [21].

In Proctor’s outcomes for implementation research, various types of stakeholders are recognized; for example, administrators, payers, health care providers, and patients or consumers and their family members, to name a few [21]. Hence, an implementation outcome, such as “acceptability,” would hold different salience for each type of stakeholder [21]. For clarity, in this review, we chose to foreground the perspectives of target users for the implementation outcome “acceptability.”

Hence, we can group the 8 outcomes proposed by Proctor et al [21] into the following three groups: (1) outcomes from a user perspective that are a function of innovation design (acceptability, adoption, appropriateness, and feasibility); (2) the implementation process (fidelity); and (3) outcomes that foreshadow embedment in routine practice from an organizational perspective (cost and cost-effectiveness, penetration, and sustainability).

#### Service Outcomes and Patient Outcomes

Service outcomes and patient outcomes indicate the effectiveness of digital health innovation in impacting service delivery or patient care and patient health or well-being, respectively. Examples of these outcomes include patient safety indicators, quantifiable health outcomes, patient satisfaction, health-related quality of life, patient empowerment, and patient knowledge.

## Results

### Search Yield

**Figure 1:**
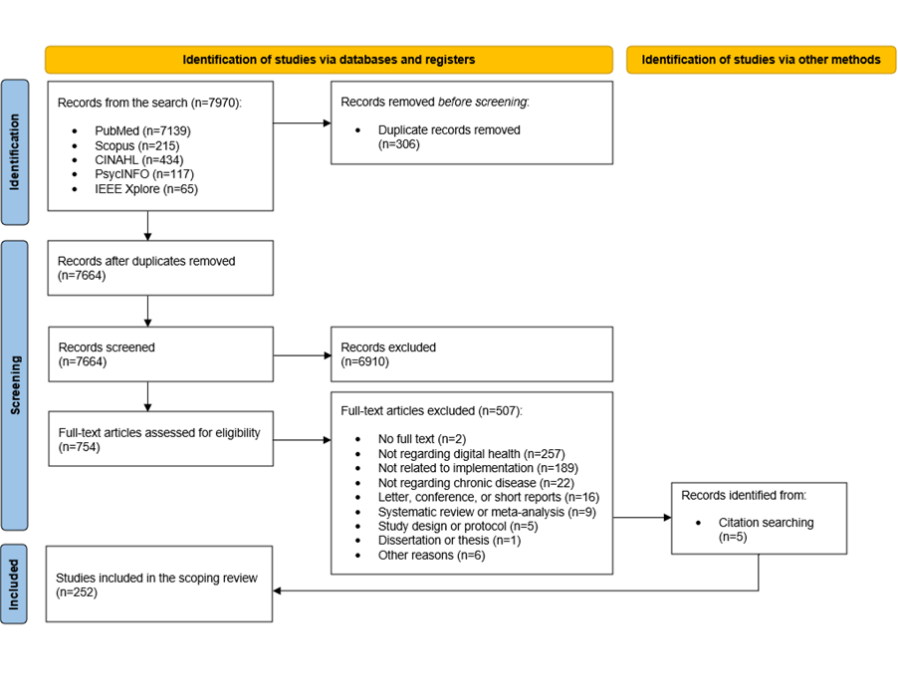
PRISMA-ScR (Preferred Reporting Items for Systematic Reviews and Meta-Analyses Extension for Scoping Review) flow diagram.

The search generated 7970 studies. After removing duplicates, 96.16% (7664/7970) studies remained. After title and abstract screening and full-text screening of 7664 and 754 studies, respectively, 3.22% (247/7664) studies remained. Manual examination of reference lists of 5 study protocols and 9 meta-analyses and systematic reviews excluded during screening yielded 5 additional studies, bringing the total number of included studies to 252 (Figure 1). The list of included studies is provided in Multimedia Appendix 1.

The most common reason for excluding full-text articles was that the innovation used was outside our scope and definition of digital health (257/507, 50.7%). Some examples include robot-assisted therapies and surgeries, minimally invasive and noninvasive medical devices, and online health education classes. Reasons for excluding full-text articles where digital health innovations were used include implementation outcomes not reported (189/507, 37.3%), disease type out of scope (22/507, 4.3%), conference proceedings or short report (16/507, 3.2%), systematic review or meta-analysis (9/507, 1.8%), study protocol (5/507, 1.0%), and dissertation (1/507, 0.2%). Other reasons for exclusion (6/507, 1.2%) were papers describing digital health implementation efforts in general and the creation of causal loop diagrams from published literature. Finally, records for which full text was not available upon reasonable search, for example, Google Scholar (Google LLC), university libraries, and contacting study authors via email or ResearchGate with no reply within 2 weeks, were excluded (2/507, 0.4%).

### Study Characteristics

Key characteristics of the included studies are presented in Table 1. The majority were single-country studies (243/252, 96.4%). Most of the studies originated from the Region of the Americas (106/252, 42.1%), the European Region (89/252, 35.3%), and the Western Pacific Region (35/252, 13.9%). About half of the included studies were published between 2015 and 2019 before the emergence of the COVID-19 pandemic (123/252, 48.8%), with the other half (129/252, 51.2%) published between 2020 and 2023, during and post–COVID-19 pandemic. The 3 most common types of chronic diseases managed were chronic respiratory diseases (64/252, 25.4%), cardiovascular diseases (33/252, 13.1%), and neurological disorders (29/252, 11.5%).

The top 3 digital health innovations implemented were mHealth (107/252, 42.5%), telehealth (97/252, 38.5%), and eHealth (61/252, 24.2%). Most innovations were intended for patients (241/252, 95.6%). The top 3 study designs were mixed methods (57/252, 22.6%), qualitative description studies (54/252, 21.4%), and randomized controlled trials (47/252, 18.7%; Multimedia Appendix 1). The sample size varied widely, ranging from 11 to as large as 23,282 (Table 2). Of the 252 studies, 82 (32.5%) reported the study’s duration, which ranged from 0.5 to 106 months, with a mean of 18.1 (SD 16.8) months.

**Table 1:** Characteristics of included studies.

Study characteristics					Papers, n (%)
**Single-country study^a^**					243 (96.4)
	Region of the Americas			106 (42.1)	
	European Region			89 (35.3)	
	Western Pacific Region			34 (13.5)	
	South-East Asia Region			9 (3.6)	
	Eastern Mediterranean Region			3 (1.2)	
	African Region			1 (0.4)	
	Unclassified geographical region: Taiwan			1 (0.4)	
**Multicountry study^a^**					9 (3.6)
	European Region			6 (2.4)	
	European Region and Region of the Americas			1 (0.4)	
	European Region, Region of the Americas, and Western Pacific Region			1 (0.4)	
	European Region and Western Pacific Region			1 (0.4)	
**Publication year**					
	Before COVID-19 pandemic, 2015-2019			123 (48.8)	
	After COVID-19 pandemic, 2020-2023			129 (51.2)	
**Type of chronic disease^b^**					
	Chronic respiratory diseases			64 (25.4)	
	Cardiovascular diseases			33 (13.1)	
	Neurological disorders			29 (11.5)	
	Diabetes			26 (10.3)	
	Chronic diseases in general			20 (7.9)	
	Mental illnesses			20 (7.9)	
	Chronic musculoskeletal diseases			19 (7.5)	
	Chronic kidney diseases			14 (5.6)	
	Hypertension			13 (5.2)	
	Cancer			12 (4.8)	
	HIV or AIDS			6 (2.4)	
	Rheumatic diseases			6 (2.4)	
	Chronic hematologic disorders			5 (2)	
	Chronic liver diseases			5 (2)	
	Chronic skin diseases			5 (2)	
	Chronic gastrointestinal diseases			4 (1.6)	
	Obesity			3 (1.2)	
	Eye diseases			2 (1.2)	
	Ear diseases			1 (0.4)	
**Type of digital health innovation^c^**					
	mHealth^d^			107 (42.5)	
	Telehealth or telemedicine			97 (38.5)	
	eHealth			61 (24.2)	
	Wearables			17 (6.7)	
	Big data or deep learning or machine learning			4 (1.6)	
	Digital health in general			1 (0.4)	
**Target user of the innovation^e^**					
	Patients			241 (95.6)	
	Physicians			58 (23)	
	Nurses			46 (18.3)	
	Allied health professionals			26 (10.3)	
	Caregivers			25 (9.9)	
	Health care professionals in general^f^			10 (4)	
	Pharmacists			5 (2)	
	Ancillary or support staff			4 (1.6)	
	Midwives			1 (0.4)	
**Framework used**					
	No framework			216 (85.7)	
	**Implementation science frameworks^g,h^**			23 (9.1)	
		Implementation theories	7 (2.8)		
		Classic theories	7 (2.8)		
		Determinant frameworks	7 (2.8)		
		Evaluation frameworks	3 (1.2)		
		Process models	1 (0.4)		
	**Other frameworks**			13 (5.2)	
		Health frameworks	7 (2.8)		
		Technology adoption frameworks	3 (1.2)		
		Frameworks developed by medical and health organizations	2 (0.8)		
		Education frameworks	1 (0.4)		
**Number of implementation strategies**					
	0			5 (2)	
	1			103 (40.9)	
	2			102 (40.5)	
	3			40 (15.9)	
	4			1 (0.4)	
	5			1 (0.4)	
**Types of outcome^i^**					
	**Implementation outcomes^j^**				
		Acceptability	171 (67.9)		
		Feasibility	38 (15.1)		
		Adoption	32 (12.7)		
		Cost^k^	6 (2.4)		
		Fidelity	4 (1.6)		
		Appropriateness	3 (1.2)		
		Sustainability	3 (1.2)		
		Penetration	1 (0.4)		
	**Patient outcomes**				
		Health outcomes	81 (32.1)		
		Satisfaction	40 (15.9)		
		Quality of life	24 (9.5)		
		Patient empowerment	3 (1.2)		
		Patient knowledge	2 (0.8)		
	**Service outcomes**				
		Safety	5 (2)		
**Assessment method used to evaluate outcome^l^**					
	Surveys			173 (68.7)	
	Interviews			95 (37.7)	
	Observations			39 (15.5)	
	Focus group discussions			18 (7.1)	
	Think-aloud protocol			7 (2.8)	

^a^Countries are categorized based on the World Health Organization’s country groupings [201].

^b^Percentages do not add up to 100% as some studies addressed >1 chronic disease.

^c^Percentages do not add up to 100% as some studies discussed >1 innovation.

^d^mHealth: mobile health.

^e^Percentages do not add up to 100% as some studies have >1 target user.

^f^The type of health care professionals is not specified.

^g^ Some studies used >1 implementation science framework.

^h^Adapted from Nilsen taxonomy for implementation theories, models, and frameworks [11].

^i^Percentages do not add up to 100% as some studies have >1 outcome.

^j^Adapted from Proctor outcomes in implementation research [21].

^k^Most studies only reported the cost of innovation; only Raeside et al [36] reported the total cost of implementation.

^l^Percentages do not add up to 100% as some studies used >1 assessment method to evaluate the outcome.

**Table 2:** Sample size of included studies.

Innovation			Sample size, n
**Single innovation**			
	Telehealth or telemedicine	23,282	
	mHealth^a^	9370	
	eHealth	5751	
	Wearables	232	
	Digital health in general	32	
	Big data or deep learning or machine learning	11	
**Multiple innovations**			
	eHealth and mHealth	9783	
	eHealth, mHealth, and wearables	457	
	mHealth and big data or deep learning or machine learning	385	
	mHealth and wearables	374	
	mHealth and telehealth or telemedicine	198	
	eHealth and telehealth or telemedicine	158	
	eHealth, mHealth, and big data or deep learning or machine learning	120	
	Telehealth or telemedicine, mHealth, and wearables	93	
	Telehealth or telemedicine, mHealth, eHealth, and big data or deep learning or machine learning	60	
	Telehealth or telemedicine and wearables	15	

^a^mHealth: mobile health.

### Definition of Digital Health

Of the 252 studies, only 24 (9.5%) included a definition of digital health. Of these 24 studies, a general definition of digital health was provided by 3 studies (12.5%): the use of ICTs for health care that includes both eHealth and mHealth [37] or as incorporating disruptive and medical technologies [38,39]. Definitions of a specific type of digital health innovation were provided by the remaining studies (21/24, 88%), of which 9 (43%) studies defined mHealth, 9 (43%) telehealth, 2 (10%) telemonitoring, and 1 (5%) defined eHealth. mHealth was defined as the use of mobile technology for services related to health care [40-48], and telehealth was defined as the use of ICT for internet-based provision of health care [49-57]. Of the 2 studies on telemonitoring, 1 (50%) described telemonitoring as an automated system [58], and the other (50%) defined it as a noninvasive patient-monitoring system [59] that uses ICT for the dissemination of patients’ clinical data from their homes to their respective health care providers [58,59]. The sole study with a definition on eHealth defined it as the use of ICT for health care [60].

### Type of Framework

Frameworks used to implement digital health innovations are presented in Table 1. Most studies (216/252, 85.7%) did not indicate or specify the framework used to guide the implementation. Of those that did, there was a good mix of implementation science (23/252, 9.1%) and other frameworks used (13/252, 5.2%).

Implementation models and theories used were the capability, opportunity, motivation, behavior (COM-B) model with the associated Behavior Change Wheel (4/252, 1.6%) and the Normalization Process Theory (3/252, 1.2%). Classic theories used were the self-determination theory (4/252, 1.6%), social cognitive theory (2/252, 0.8%), and Bandura’s self-efficacy theory (1/252, 0.4%). The determinant frameworks used were the Consolidated Framework for Implementation Research (4/252, 1.6%); Exploration, Preparation, Implementation, and Sustainment framework (1/252, 0.4%); the integrated Promoting Action on Research Implementation in Health Services framework (1/252, 0.4%); and the Theoretical Domains Framework (1/252, 0.4%). The sole process framework used was the model by Grol and Wensing [203] (1/252, 0.4%), although the Exploration, Preparation, Implementation, and Sustainment framework arguably straddles both determinant and process frameworks and could be included here.

### Implementation Strategies

Most studies used 1 (103/252, 40.9%) or 2 (102/252, 40.5%) strategies to implement digital health (Table 1). Table 3 shows the implementation strategies used, mapped to the ERIC taxonomy [202,204]. The top 3 strategies used in terms of frequency were collecting feedback from target users (ERIC 46: obtain and use patients or consumers and family feedback; 196/252, 77.8%); reviewing clinical performance details and providing feedback (ERIC 5: audit and provide feedback; 106/252, 42.1%); and conducting pilot studies before implementation (ERIC 61: stage implementation scale-up; 85/252, 33.7%).

**Table 3:** Implementation strategies (N=252).

Implementation strategy	Papers, n (%)^a^
ERIC^b^ 46: obtain and use patients or consumers and family feedback	196 (77.8)
ERIC 5: audit and provide feedback	106 (42.1)
ERIC 61: stage implementation scale-up	85 (33.7)
ERIC 41: involve patients or consumers and family members	19 (7.5)
ERIC 4: assess for readiness and identify barriers and facilitators	10 (4)
ERIC 17: conduct local consensus discussions	3 (1.2)
ERIC 18: conduct local needs assessment	3 (1.2)
ERIC 56: purposefully re-examine the implementation	3 (1.2)
ERIC 19: conduct ongoing training	2 (0.8)
ERIC 35: identify and prepare champions	2 (0.8)
ERIC 55: provide ongoing consultation	2 (0.8)
ERIC 64: use advisory boards and workgroups	2 (0.8)
ERIC 65: use an implementation adviser	2 (0.8)
ERIC 69: use mass media	2 (0.8)
ERIC 15: conduct educational meetings	1 (0.4)
ERIC 26: develop and implement tools for quality monitoring	1 (0.4)
ERIC 27: develop and organize quality monitoring systems	1 (0.4)
ERIC 29: develop educational materials	1 (0.4)
ERIC 54: provide local technical assistance	1 (0.4)
ERIC 63: tailor strategies	1 (0.4)
ERIC 67: use data experts	1 (0.4)

^a^Percentages do not add up to 100% as some studies used >1 implementation strategy.

^b^ERIC refers to the Expert Recommendations for Implementing Change taxonomy for implementation strategies.

### Outcomes in Implementation Research

#### Overview

The main assessment method used to evaluate outcomes was surveys (173/252, 68.7%; Table 1). Outcome measures used to evaluate the implementation and effectiveness of digital health innovations were sorted into 3 categories, following Proctor et al [21] (Table 1 and Multimedia Appendix 1): implementation outcomes, service outcomes, and patient outcomes. While favorable implementation outcomes indicate successful implementation, the effectiveness of the digital health innovation is determined by service and patient outcomes. The 3 most common outcomes measured were acceptability (171/252, 67.9%), which is an implementation outcome, and 2 patient outcomes, namely health outcomes (81/252, 32.1%) and patient satisfaction (40/252, 15.9%). Service outcomes were largely not measured, with only a handful of studies (5/252, 2%) monitoring safety or adverse events.

#### Implementation Outcomes

Most studies that measured the acceptability, feasibility, and adoption of eHealth found it acceptable (38/39, 97%), feasible (7/10, 70%), and with a high rate of adoption (10/10, 100%). The sole study on eHealth that assessed fidelity reported that it was high among target users [61]. Similarly, most studies that evaluated the acceptability, feasibility, and adoption of mHealth found it to be acceptable (70/74, 95%), feasible (16/18, 89%), and with a high rate of adoption (14/15, 93%). However, the 2 studies that investigated the appropriateness of mHealth reported that it was rated low by target users [42,62].

Likewise, most studies that assessed the acceptability and feasibility of telehealth found it acceptable (59/61, 97%) and feasible (13/14, 93%). The studies that measured the adoption of telehealth (4/5, 80%) reported that the adoption rate was high. All the studies that evaluated the fidelity of telehealth concluded that it was high among target users (3/3, 100%), and the 2 studies that analyzed the appropriateness of telehealth found it appropriate [56,58]. However, the sole study on telehealth that measured penetration observed that it varied widely across different health systems [63], and the 2 studies on telehealth that assessed sustainability received mixed reviews [63,64].

As for wearables, most studies (7/8, 88%) that evaluated its acceptability found it acceptable. The sole study focusing on wearables that measured feasibility concluded that it was feasible [65], and the 2 studies that assessed the adoption reported that the adoption rate was high (Multimedia Appendix 1) [66,67].

#### Service Outcomes

Most studies (247/252, 98%) did not measure service outcomes, such as efficiency, safety, effectiveness, equity, patient-centeredness, and timeliness. Of the studies that did (5/252, 2%), study authors measured safety and concluded that there were no major adverse incidents caused by the innovation [68-71] or that the adverse events only occurred in a small number of users [72].

#### Patient Outcomes

Of 252 studies, 89 (35.3%) measured patients’ health or quality of life. Most of these studies (65/89, 73%) reported an improvement in at least 1 aspect of patients’ health or quality of life after the implementation of eHealth, mHealth, or telehealth, although 1 study (1%) saw a drop in patients’ health status but an improvement in quality of life. Of the 24 remaining studies, 22 observed no substantial difference (22/89, 25%), while 2 noticed a reduction in patients’ health status (2/89, 2%).

Of 252 studies, 40 (15.9%) measured patient satisfaction with the innovation. Most of these studies (39/40, 98%) reported that patient satisfaction with eHealth, mHealth, and telehealth was generally high. Improvements in patient empowerment, specifically self-management and self-care were seen after the implementation of eHealth and mHealth innovations, respectively [73-75]. In addition, improvements in patient knowledge were seen after the implementation of eHealth [76,77]. None of the articles on wearables investigated patient outcomes (Multimedia Appendix 1).

### Barriers and Enablers to Implementation

Of the 252 studies, 123 (48.8%) addressed barriers and enablers to implementation. Most of these studies elicited barriers and facilitators as part of the study aims (110/123, 89.4%), with the exception of 13 studies (10.6%) that reported barriers and facilitators as part of the findings or discussion. The barriers and enablers have been grouped into 4 categories: external factors, factors related to health care workers, patient-related factors, and factors pertinent to both patients and health care workers.

The categories were generated based on the barriers and enablers identified in this review. Barriers and enablers related to recipients (target users) of the digital health innovations were categorized as “factors related to health care workers,” “patient-related factors,” or “factors pertinent to both patients and health care workers.” Barriers and enablers related to systems, policies, and infrastructure were categorized as “external factors.”

### Barriers

#### Overview

**Figure 2:**
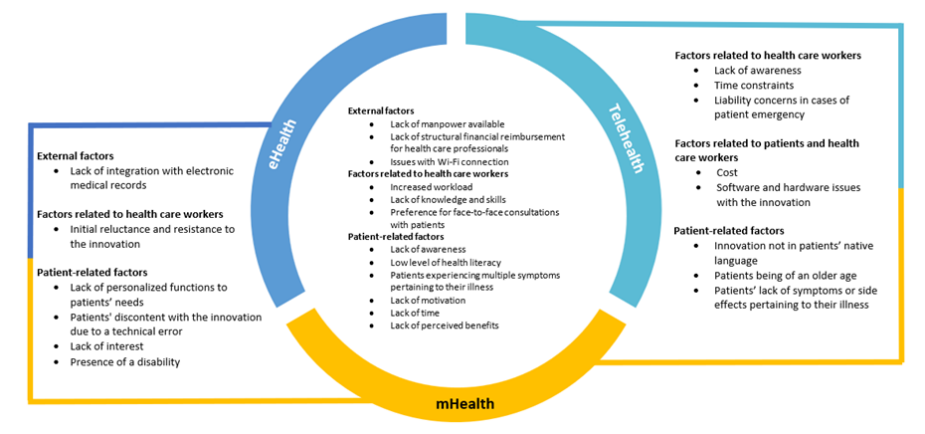
Common barriers affecting the implementation of eHealth, mobile health (mHealth), and telehealth.

Common barriers affecting the implementation of eHealth, mHealth, and telehealth are presented in Figure 2, with specific barriers for the individual innovations detailed in Table 4.

**Table 4:** Specific barriers pertaining to individual innovations.

Innovation	External factors	Factors related to health care workers	Factors related to patients and health care workers	Patient-related factors
Digital health in general^a^	Lack of resources to manage the data generated by the digital health tool Poor quality of health data collected	Lack of strong evidence for health care workers to safely implement digital health	—^b^	—
eHealth	Lack of resources to manage privacy and safety concerns	Lack of access to eHealth tool Cost Initial skepticism pertaining to the innovation Concerns about the eHealth tool’s reliability	Insufficient support and feedback provided by health care workers to patients Lack of ease of use	Difficulties in loading and logging into the eHealth portal Patients being reminded of being sick when using eHealth Patients experiencing unexpected life events Patients not being able to use their own personal device as the eHealth tool Lack of training and support provided
mHealth^c^	Lack of support from senior physicians at the initial stages of implementation Lack of systematic assessment and reporting of patients’ health records or lack of integrated care for patients Mismatch between the desire of the clinic director to implement mHealth and the buy-in from staff carrying out the implementation	Time constraints when mHealth was first implemented	—	High level of fatigue Lack of access to smartphones Presence of too many tasks to complete on the mHealth app Preference or switching to nontraditional medicine
Telehealth	—	Difficulties assessing patients via telehealth Lack of buy-in and engagement Lack of communication among the different health care professionals	Lack of technical support Lack of space and telehealth equipment	Lack of family support
Wearables	Technical difficulties linking wearable to a web-based server	—	—	Discomfort from using wearables Lack of trust Poor rapport with clinicians

^a^Type of digital health innovation not specified.

^b^Not applicable.

^c^mHealth: mobile health.

#### Common Barriers Affecting Implementation

Data privacy emerged as the foremost common barrier among various digital health innovations [37,38,43,45,78-92]. Patient preference for in-person consultations [53,66,83,84,89,93-95] and their level of comfort with digital health or technology in general [66,96-99] were common barriers to the implementation of eHealth, mHealth, telehealth, and wearables. The lack of manpower [63,80,85,100-104] and the preference of health care workers for in-person consultations with patients [43,60,78,85,90,105-107] were shared barriers for eHealth, mHealth, and telehealth innovations. The lack of integration with electronic medical records [60,80,81,90,108-113] was a reported barrier for both eHealth and mHealth. Health care workers’ time constraints were an important barrier to the implementation of both mHealth and telehealth [100,104,114,115].

#### Barriers Unique to Type of Technology

Some barriers unique to eHealth included the lack of ease of use for both patients and health care workers [79,91] and the initial skepticism of health care workers about eHealth [81]. Barriers distinctive to mHealth were the lack of support from senior management at the initial phase of implementation [101] and patients’ lack of access to smartphones [40,96]. For telehealth, unique barriers included health care workers facing difficulties evaluating patients over the internet [104] and the lack of space and equipment for patients and health care workers to attend and conduct telehealth visits, respectively [63,83,98,104,105,115,116]. Barriers unique to wearables include the presence of discomfort after wearing the item [41] and patients’ lack of trust in the technology [65]. For the sole study that evaluated digital health in general, some unique barriers were the lack of resources to handle the information collected by digital health tools and the lack of solid evidence to implement digital health safely [37] (Table 4).

### Enablers

**Figure 3:**
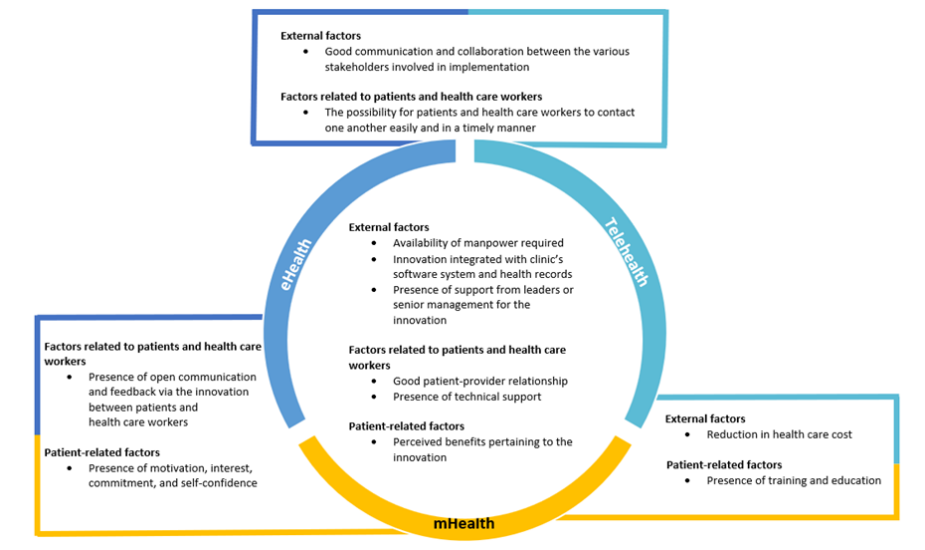
Common enablers affecting the implementation of eHealth, mobile health (mHealth), and telehealth.

Common enablers affecting the implementation of eHealth, mHealth, and telehealth are depicted in Figure 3, while specific enablers of each type of innovation are presented in Table 5.

**Table 5:** Specific enablers pertaining to individual innovations.

Innovation	External factors	Factors related to health care workers	Factors related to patients and health care workers	Patient-related factors
Digital health in general^a^	—^b^	High level of digital literacy	—	—
eHealth	—	Awareness of eHealth Positive perception of eHealth Perception of having control over patient management Increased productivity Presence of incentives	—	Ability to engage and use the innovation
mHealth^c^	—	—	mHealth app having a comprehensive set of features Having a say in the development of mHealth Presence of health care workers monitoring patients’ use of mHealth	Being of an older age Familiarity with smartphone High level of self-efficacy Perception that the innovation is credible Presence of reminders to use mHealth
Telehealth	Presence of adequate funding needed for the implementation Telehealth as a complement, not a replacement of in-person consultations	Presence of engagement with health care workers on telehealth Perception that telehealth is credible High confidence level with regard to using telehealth Presence of administrative support	—	High confidence and trust in the health care system Presence of a program champion Presence of encouragement from health care workers to patients to adopt telehealth Presence of flexibility to attend telehealth sessions at a preferred place and time Presence of social support Comfort with telehealth
Wearables	—	—	—	Being of a younger age Innovation easily worn under clothing without snagging Presence of severe symptoms

^a^Type of digital health innovation not specified.

^b^Not applicable.

^c^mHealth: mobile health.

#### Common Enablers Affecting Implementation

The common enablers between eHealth, mHealth, telehealth, and digital health in general were the presence of training for health care workers [37,45,60,78,80,100,101,117] and personalization of digital health features to patients’ needs [37,42,58,80,82,115,118-121,205]. The sole common enabler among eHealth, mHealth, telehealth, and wearables was the ease of use of the respective technologies [36,43,60,63,66,78,80,82,84,87,96,108,109,111,115,118,121,205-212]. With regard to eHealth, mHealth, and telehealth, some common enablers among them included a good relationship between the patient and the health care worker [79,104,118,205,213,214] and the availability of manpower to implement the innovation [98,101,215]. The presence of motivation to use the innovation was particular to both eHealth and mHealth [119,206]. The presence of education and training for patients was an enabler for both mHealth and telehealth [43,45,78,87,206]. Good communication between the implementation stakeholders was an enabler for both telehealth and eHealth [58,102,104,105,108,114,215].

#### Enablers Unique to Type of Technology

Enablers unique to eHealth included the ability of health care workers to evaluate more patients without an increase in time commitment [90,216] and the presence of incentives for health care workers, such as the provision of financial reimbursement and continuing medical education points [108]. Enablers distinctive to mHealth included patients’ familiarity with smartphones [217] and a high level of self-efficacy [216]. For telehealth, unique enablers included the presence of encouragement from health care workers to patients to use telehealth [98,114,218] and flexibility for patients to attend telehealth consultations at their convenience [83,85,93,105,114]. With regard to wearables, examples of unique enablers were the ability to wear the innovation easily under clothing and users being of a younger age group, as wearable designs often do not consider the requirements and needs of older adults [66]. For the sole study that evaluated digital health in general, a high level of digital literacy among health care workers was a specific enabler [37] (Table 5).

### Lessons Learned Shared by Study Authors

Of the 252 studies, 62 (24.6%) specified lessons learned. Many patients are generally open to digital health [207], and the initial uptake is usually high [219]. However, health care workers often perceive it as an increase in workload [110]. For digital health innovations to be successful, it is important for them to be tailored to the patient’s needs [37,41,67,83,84,86,87, 89,111,115,119,121,212,220-227], to enhance the patient-provider relationship [90], and to complement physical appointments [57,105,222,228]. Effective communication between implementation stakeholders is required [87,98,229], and ongoing evaluation is needed to ensure that the uptake remains high [60]. In addition, the benefits of digital health need to be evident to target users [230].

To increase the uptake of digital health, support from relevant authorities is needed [109], along with raising awareness of its benefits [45,78] and embedding it into existing workflows [60,90]. Other ways to increase uptake include the provision of resources required for implementation [98,225,231], the presence of technical support [37,60,225,231], and training for target users [60,213,225]. In addition, digital health innovations should be user-friendly [41,45,219,231,232], target users should be included in its development and implementation [60,66,110,221,233,234], and a transition stage should be included for target users to adapt to the innovation [60].

### Recommendations Shared by Study Authors

Of the 252 studies, 122 (48.4%) studies shared recommendations. Study authors recommended conducting a study of a longer duration [37,51,230,235-240] or conducting a larger study [74,96,120,210,224,232,241-244] for future studies. Many authors also recommended using controlled trials for future studies [54,97,110,112,113,207,208, 211,230,231,245-256]. Some suggested larger or long-term trials [97,110,112,113,207,211,245,246,248,250,253,255-257], while others proposed clinical trials in general [258,259] or having a control group [46,56,96,260-263].

Furthermore, some authors recommended future research in other contexts, such as different countries or cities [43,59,68,88], different health care systems [59,68] or settings [47,211,264-266], different stakeholder groups involved in the implementation [43,80,96,267], different demographics in terms of age [268] or socioeconomic status [222,269], more diverse demographics [89,91,104,107,110,232,243, 257,258,264,266,270], different illnesses [271-274], or severity of disease [99]. In contrast, other authors suggested focusing on patients with similar illness classification [242,275].

Other proposals pertinent to implementation include using different implementation strategies [76], monitoring different outcomes [36,107,225,276], collecting outcomes more frequently [277], or understanding the association between different features of the innovation and their corresponding outcomes [55,278,279]. Other suggestions include personalizing the innovation to individual users [88,95,121,258,280], having more extensive user testing [115,273,281], or making relevant changes to current features of the innovation based on the research findings [213,219,241,282,283].

Moreover, several authors recommended focusing on efficacy [43,45,49,70,217,247,256,284-287], validation [229], effectiveness [65,210,221,258,288-290], cost-effectiveness [45,96,115,247,281], clinical applicability [291], or usability of the innovation [292]. Other proposals include using a mixed methods approach for future studies [43,45,218,248,293] or qualitative research methods to understand target users’ experiences with digital health [42,43,45,59,230].

## Discussion

### Principal Findings

This review bridges a critical gap in the deployment of digital health innovations for the management of chronic diseases by eliciting how such innovations have been implemented and evaluated to date. We conducted the review through the lens of implementation science to generate actionable findings for future real-world implementation of digital health innovations.

First, >90% of the 252 studies included in this review did not report using an implementation science framework for planning, guiding, or evaluating the real-world deployment of digital health technologies, despite the availability of many suitable frameworks to guide implementation [11]. This reportedly low use of implementation science frameworks may be due to implementation science being an emerging field. Hence, awareness and the potential usefulness of such frameworks for implementing digital health technologies are not yet widespread. Of the studies that reported using an implementation science framework, implementation theories, classic theories, and determinant frameworks were the most frequently used. Implementation science frameworks developed specifically for the deployment of digital health technologies were rarely used; for example, only 3 studies used the Normalization Process Theory and none used the Nonadoption, Abandonment, Scale-up, Spread, and Sustainability framework [294].

Of 252 studies, only 23 (9.1%) leveraged an implementation science framework. Study authors used these frameworks to underpin qualitative data collection, data analysis, or interpretation of findings; to inform the design of behavior change and implementation strategies to enable successful deployment; and for the evaluation of implementation outcomes. Although most included studies (229/252, 90.9%) did not formally use implementation science frameworks to guide implementation, these studies have, nevertheless, addressed or incorporated some elements of these frameworks. For example, 48.8% (123/252) of the studies assessed barriers and enablers affecting implementation. The low use of implementation science frameworks represents missed opportunities to generate reliable translation and scale-up of evidence-based innovations through a deeper understanding of contextual influences and eliciting mechanisms of implementation, as well as productive theorizing of implementation research [23,295].

Second, of the studies that measured implementation or patient outcomes, most (188/227, 82.8%) reported positive implementation or patient outcomes despite the low use of implementation science frameworks in the deployment of digital health innovations. Target users generally found digital health to be acceptable and feasible, and study authors reported high adoption. Only 6 (2.4%) of the 252 studies included appropriateness and sustainability as outcome measures to evaluate implementation, and the results were mixed. Of the studies that measured patients’ health outcomes or quality of life, 77% (64/83) reported improvements, with patients being generally satisfied with digital health. These outcomes were mainly assessed using surveys (173/252, 68.7%); this is unsurprising as surveys are commonly used in health services research [296]. Most surveys contained at least 1 validated scale to assess outcomes (103/173, 59.5%) or were created by study authors (85/173, 49.1%).

Third, the most commonly used implementation strategies were evaluative and iterative strategies as per the ERIC taxonomy [202]; this is not surprising, given the relatively higher importance and feasibility of these strategies [202]. Studies focused on innovation development used a variety of co-design strategies with target users, such as patients and health care professionals. Strategies such as obtaining user feedback and conducting pilots before implementation were necessary, considering that most of the digital health innovations were in the early stages of real-world deployment.

### Comparison to Prior Work

Overall, the findings from this review are similar to previous scoping reviews in emphasizing the usefulness of digital health innovations, and they are viewed positively by target users [297-299]. Willis et al [297] observed that many included studies found statistically significant improvements in implementation outcomes (eg, adoption and acceptability) and health care performance outcomes (eg, validated health measures), congruent with the findings of our review. Patel et al [298] highlighted that numerous studies indicated that many participants were willing to use digital health innovations, especially if they are personalized to target users’ needs and preferences, which is a key enabler found in our review. Likewise, Lim et al [299] argued that most participants found digital health innovations highly acceptable and perceived them to be convenient, user-friendly, and practical.

Despite the above, many studies in this review indicated numerous barriers that could affect implementation. Notable barriers include concerns over data privacy [37,38,43,45,78-92] and patients’ preference for physical consultations [53,55,66,83,84,89,93-95]. While it remains to be seen whether the convenience and flexibility of accessing health care via digital modalities would trump patient preference for physical consultations, assuming the quality of care is not compromised, ensuring data privacy is nonnegotiable. Not being able to assure data privacy is arguably the most formidable barrier affecting successful implementation [300,301].

In our review, two-thirds (171/252, 67.9%) of the included studies examined acceptability as an implementation outcome, followed by feasibility (38/252, 15.1%), and adoption (32/252, 12.7%). Other implementation outcomes, such as penetration, sustainability, appropriateness, fidelity, and cost, were infrequently reported (number of studies ranged between 1 and 6 studies). Similarly, a recent scoping review by Proctor et al [302] investigating the progress of implementation outcomes research found that 52.5% (210/400) of their included studies examined acceptability [302]. Fidelity was the next most commonly examined outcome (157/400, 39.3%), followed by feasibility (154/400, 38.5%), adoption (106/400, 26.5%), and appropriateness (87/400, 21.8%). Implementation outcomes such as penetration (64/400, 16%), sustainability (63/400, 15.8%), and cost (31/400, 7.8%) were relatively less frequently examined [302], albeit in higher proportions of included studies compared to our review. Our finding that service outcomes were rarely reported (5/252, 2%) contrasts with that reported in the scoping review by Proctor et al [302], where a small percentage (22/400, 5.5%) of included studies not only reported service outcomes but also examined the relationship between implementation outcomes and service outcomes.

### Limitations and Strengths

There are several limitations to this review. First, the identification and classification of implementation strategies and outcomes for included studies were not without challenges, as studies used various terms to describe these constructs. To reduce errors and to ensure consistency in the interpretation of terms for the purposes of data extraction, we piloted the data extraction form and resolved discrepancies before actual extraction.

Second, we were not able to judge how well implementation strategies were carried out, regardless of whether an implementation science framework was used or not, apart from documenting the outcomes reported by the study authors. We cannot rule out that in some included studies, the outcomes were suboptimal due to poorer execution of implementation strategies rather than the lack of an implementation science framework to underpin the work per se.

Third, we did not assess the quality of reporting against the Standards for Reporting Implementation Studies (StaRI) [303], as not all included studies explicitly claimed to be an implementation study. StaRI comprises a 27-item checklist spanning both the implementation strategy and the clinical, health care, or public health intervention (the innovation) being implemented. According to StaRI, study authors should describe the scientific background and explain their rationale for selecting the underpinning implementation theory, model, or framework and implementation strategies. Study authors should do the same for the innovation being implemented and include a description of the evidence of its effectiveness [303]. Adhering to StaRI would also require a description of how the “selected strategy is expected to achieve its effects”; for example, a logic model or pathway showing hypothesized mechanisms of action for how each implementation strategy is expected to bring about desired patient outcomes [303]. The logic model or pathway does not replace the use of an implementation theory, model, or framework to underpin the work of implementation.

Fourth, we did not include preprints or unpublished literature, which might have affected our findings. Digital health is a rapidly growing field, and pioneering innovations may have yet to be published in peer-reviewed journals. However, preprints are more likely to capture the development and validation phases of digital health innovations rather than last-mile real-world implementation, which is the scope of this review.

Strengths of this review include a comprehensive search strategy and broad inclusion criteria to ensure that we capture as many relevant studies as possible. The included studies are not limited to a particular geographical region or type of digital health innovation, thus allowing a representative overview to be generated. We categorized the findings on barriers and enablers to understand differences related to patients, health care workers, and external factors across different digital health innovations, which will be useful for planning and designing future implementation studies.

### Future Directions

This scoping review generated a summary of how digital health in chronic disease management is currently implemented. The benefit of using implementation science frameworks was not unequivocal and was out of the scope of this review. Nevertheless, our findings established a basis for future studies to investigate whether using an implementation science framework (and how well it is used) compared to not using one would yield better or more consistent outcomes. In addition, we agree with Proctor et al [302] that future studies testing the relationships between implementation strategies and implementation, service, and patient outcomes are needed.

The digital health innovation being implemented should not unduly burden target users, whether patients or health care providers. Investing in co-design with target users as early as practicable is a smart way to ensure that the innovation is fit for purpose and to pre-emptively identify user issues. However, as users and contexts continually evolve and change, acceptability of digital health innovation may change with time. Hence, the acceptability of the innovation should be assessed at several time points, and the findings should be used to inform iterative improvements in innovation design.

The process of deployment is as important as the features of the innovation. Target users require adequate preparation and support throughout the implementation. It should be ensured that process measures and adverse events are monitored consistently and addressed. Planning for and monitoring sustainability should be a key outcome measure, given the not-insignificant investment of resources in digital health.

### Conclusions

Digital health has changed how health care is viewed and managed; nonetheless, how it is implemented in real-world settings can be further optimized. This implementation science–guided scoping review generated a comprehensive summary of the various ways digital health innovations have been implemented and evaluated for chronic disease management. Findings serve as a useful resource for physicians, researchers, health system managers, and policy makers when designing the successful implementation of digital health innovations.

## Appendix

Multimedia Appendix 1 Supplementary materials which include the PRESS (Peer Review of Electronic Search Strategies) checklist, search strategies, data extraction, list of studies in the scoping review, types of study, and the outcome measures used to evaluate implementation success and effectiveness of the digital health innovation.

Multimedia Appendix 2 PRISMA-ScR Checklist.

## Abbreviations

COM-B: capability, opportunity, motivation, behavior
ERIC: Expert Recommendations for Implementing Change
ICT: information and communication technology
mHealth: mobile health
PRESS: Peer Review of Electronic Search Strategies
StaRI: Standards for Reporting Implementation Studies
