# Supplementary material for: Current Implementation of Digital Health in Chronic Disease Management: Scoping Review
Source: J Med Internet Res. 2024 Dec 12;26:e53576. doi: 10.2196/53576 (PMC11671791; doi:10.2196/53576)
Supplement: Multimedia Appendix 1 [file jmir_v26i1e53576_app1.docx]

**Current Implementation of Digital Health in Chronic Disease Management: Scoping Review**

Candelyn Pong^1^, Rachel Marjorie Wei Wen Tseng^2,3^, Yih Chung Tham^2,4^, Elaine Lum^1,5*^

^1^Health Services and Systems Research, Duke-NUS Medical School, National University of Singapore, Singapore.

^2^Singapore Eye Research Institute, Singapore National Eye Centre, Singapore.

^3^Duke-NUS Medical School, National University of Singapore, Singapore.

^4^ Centre for Innovation and Precision Eye Health & Department of Ophthalmology, Yong Loo Lin School of Medicine, National University of Singapore, Singapore.

^5^Centre for Population Health Research and Implementation, SingHealth, Singapore.

*Corresponding author

Supplementary Materials:

[Table S1. PRESS (Peer Review of Electronic Search Strategies) checklist 2](#_Toc183544327)

[S2. Search strategies 5](#_Toc183544328)

[PubMed 5](#_Toc183544329)

[Scopus 6](#_Toc183544330)

[CINAHL 7](#_Toc183544331)

[PsycINFO 8](#_Toc183544332)

[IEEE Xplore 9](#_Toc183544333)

[S3. Data extraction 10](#_Toc183544334)

[S4. List of studies in scoping review 11](#_Toc183544335)

[S5. Type of study 25](#_Toc183544336)

[S6. Outcome measures used to evaluate implementation success and effectiveness of the digital health innovation 26](#_Toc183544337)

# Table S1. PRESS (Peer Review of Electronic Search Strategies) checklist

| **Translation of the research question** | Does the search strategy match the research question/PICO? | Yes, matches the research question |
| --- | --- | --- |
|  | Are the search concepts clear? | Yes |
|  | Are there too many or too few PICO elements included? | No |
|  | Are the search concepts too narrow or too broad? | No |
|  | Does the search retrieve too many or too few records? (Please show number of hits per line.) | No |
|  | Are unconventional or complex strategies explained? | Not applicable |
| **Boolean and proximity operators (these vary based on search service)** | Are Boolean or proximity operators used correctly? | Yes |
|  | Is the use of nesting with brackets appropriate and effective for the search? | Yes |
|  | If NOT is used, is this likely to result in any unintended exclusions? | Not applicable |
|  | Could precision be improved by using proximity operators (e.g., adjacent, near, within) or phrase-searching instead of AND? | No |
|  | Is the width of proximity operators suitable (e.g., might adj5 pick up more variants than adj2)? | Not applicable |
| **Subject headings (database-specific)** | Are the subject headings relevant? | Yes |
|  | Are any relevant subject headings missing; e.g., previous index terms? | No |
|  | Are any subject headings too broad or too narrow? | No |
|  | Are subject headings exploded where necessary and vice versa? | Yes |
|  | Are major headings (“starring” or restrict to focus) used? If so, is there adequate justification? | No |
|  | Are subheadings missing? | No |
|  | Are subheadings attached to subject headings? (Floating subheadings may be preferred.) | Yes |
|  | Are floating subheadings relevant and used appropriately? | Not applicable |
|  | Are both subject headings and terms in free text (see below) used for each concept? | Yes |
| **Text word searching (free text)** | Does the search include all spelling variants in free text (e.g., UK versus US spelling)? | Yes, for selected databases only. This is because certain databases such as PubMed and Scopus automatically include both the US and UK spelling in the search by default. |
|  | Does the search include all synonyms or antonyms (e.g., opposites)? | Synonyms are included, antonyms are not. |
|  | Does the search capture relevant truncation (i.e., is truncation at the correct place)? | Yes |
|  | Is the truncation too broad or too narrow? | No |
|  | Are acronyms or abbreviations used appropriately? | Not applicable |
|  | Do they capture irrelevant material? Are the full terms also included? | Irrelevant words/phrases are not included in the search. Full terms are included. |
|  | Are the keywords specific enough or too broad? Are too many or too few keywords used? Are stop words used? | The keywords are sufficient and specific enough. We did not use any stop words. |
|  | Have the appropriate fields been searched; e.g., is the choice of the text word fields (.tw.) or all fields (.af.) appropriate? Are there any other fields to be included or excluded (database-specific)? | Yes, appropriate fields have been searched. There are no other fields to be included or excluded. |
|  | Should any long strings be broken into several shorter search statements? | No |
| **Spelling, syntax and line numbers** | Are there any spelling errors? | No |
|  | Are there any errors in system syntax; e.g., the use of a truncation symbol from a different search interface? | No |
|  | Are there incorrect line combinations or orphan lines (i.e., lines that are not referred to in the final summation that could indicate an error in an AND or OR statement)? | No |
| **Limits and filters** | Are all limits and filters used appropriately and are they relevant given the research question? | Yes |
|  | Are all limits and filters used appropriately and are they relevant for the database? | Yes |
|  | Are any potentially helpful limits or filters missing? Are the limits or filters too broad or too narrow? Can any limits or filters be added or taken away? | No |
|  | Are sources cited for the filters used? | Yes. We provided a citation for our justification for the date range used. |

# S2. Search strategies

## PubMed

Search conducted on March 31, 2023

| **Query** | **Search String** | **Results retrieved** |
| --- | --- | --- |
| #1 | (Digital health OR digital health technology OR Mobile health OR telehealth OR telemedicine OR digital care programs OR health technologies OR eHealth OR mHealth OR digital healthcare OR wearable medical device OR mobile medical applications OR mobile medical software OR mobile health application OR ((health) AND (wearable devices OR wearables OR wearable technology OR deep learning OR machine learning OR artificial intelligence OR human-centered ai OR human-centered computing OR big data)) OR ("Telemedicine"[MAJR] OR "Telemedicine"[MeSH] OR "Telemedicine/standards"[MAJR] OR ("Digital Technology"[MAJR] OR "Digital Technology/instrumentation"[MAJR] OR "Digital Technology/methods"[MAJR] OR "Wearable Electronic Devices"[MeSH] OR "Mobile Applications/standards"[MAJR] OR "Mobile Applications"[MeSH] OR "Artificial Intelligence/trends"[MeSH] OR "Information Technology"[MAJR] OR "Machine Learning"[MeSH] OR "Deep Learning"[MeSH] OR "Artificial Intelligence"[MeSH] OR "Big Data"[MeSH]) AND "health"[MeSH])) | 663,515 |
| #2 | (implementation OR translation OR translational OR dissemination OR evaluation) OR ("Translational Medical Research"[MeSH] OR "Implementation Science"[MeSH]) | 6,091,382 |
| #3 | Long-term illness OR Long-term disease OR Long-term health condition OR Chronic illness OR Chronic disease OR Chronic condition OR Chronic disorder OR Chronic health OR Life-long disease OR Life-long condition OR Life-long illness OR "Chronic Disease"[Mesh] | 1,562,114 |
| #4 | #1 AND #2 AND #3 | 18,312 |
| #5 | #4 NOT (review OR (systematic review)) | 13,184 |
| #6 | Limit to articles with full text | 12,760 |
| #7 | Limit to articles on humans | 9,185 |
| #8 | Limit to articles in English | 8,995 |
| #9 | Limit to articles published from January 2015 to present | 7,139 |

## Scopus

Search conducted on March 31, 2023

| **Query** | **Search String** | **Results retrieved** |
| --- | --- | --- |
| #1 | ( TITLE-ABS-KEY ( "Digital health" OR "digital health technology" OR "Mobile health" OR "telehealth" OR "telemedicine" OR "digital care programs" OR "health technologies" OR "eHealth" OR "mHealth" OR "digital healthcare" OR "wearable medical device" OR "mobile medical applications" OR "mobile medical software" OR "mobile health application" ) ) OR ( ( TITLE-ABS-KEY ( "health" ) ) AND ( TITLE-ABS-KEY ( "wearable devices" OR "wearables" OR "wearable technology" OR "deep learning" OR "machine learning" OR "artificial intelligence" OR "human-centered ai" OR "human-centered computing" OR "big data" ) ) ) | 287,133 |
| #2 | TITLE-ABS-KEY ( "implementation" OR "translation" OR "translational" OR "dissemination" OR "evaluation" ) | 6,600,124 |
| #3 | TITLE-ABS-KEY ( "Long-term illness" OR "Long-term disease" OR "Long-term health condition" OR "Chronic illness" OR "Chronic disease" OR "Chronic condition" OR "Chronic disorder" OR "Chronic health" OR "Life-long disease" OR "Life-long condition" OR "Life-long illness" ) | 451,213 |
| #4 | #1 AND #2 AND #3 | 2,534 |
| #5 | #4 AND NOT (review OR ("systematic review")) | 405 |
| #6 | Limit to articles in English | 371 |
| #7 | Exclude articles on Veterinary | 370 |
| #8 | Limit to articles published from January 2015 to present | 215 |

## CINAHL

Search conducted on March 31, 2023

| **Query** | **Search String** | **Results retrieved** |
| --- | --- | --- |
| S1 | ( "digital health" OR (MH "Telehealth+") OR "telehealth" OR "mobile health" OR "digital health technology" OR (MH "Telemedicine+") OR "telemedicine" OR "digital care programs" OR "digital care programmes" OR "health technologies" OR "eHealth" OR "mHealth" OR "digital healthcare" OR "wearable medical device" OR "mobile medical applications" OR "mobile medical software" OR "mobile health applications ) OR ( ( (MH "Health+") OR "health" ) AND ( "wearable devices" OR (MH "Wearable Sensors+") OR "wearables" OR "wearable technology" OR (MH "Deep Learning+") OR "deep learning" OR (MH "Machine Learning+") OR "machine learning" OR (MH "Artificial Intelligence+") OR "artificial intelligence" OR "human-centered ai" OR "human-centered computing" OR (MH "Data Analytics+") OR "big data" ) ) | 75,359 |
| S2 | (MH "Implementation Science") OR "implementation" OR "translation" OR (MH "Translational Medical Research") OR "translational" OR "dissemination" OR (MH "Evaluation") OR "evaluation" | 1,262,732 |
| S3 | "long-term illness" OR "long-term disease" OR "long-term health condition" OR "chronic illness" OR (MH "Chronic Disease") OR "chronic disease" OR "chronic condition" OR "chronic disorder" OR "chronic health" OR "life-long disease" OR "life-long condition" OR "life-long illness" | 95,878 |
| S4 | S1 AND S2 AND S3 | 808 |
| S5 | S4 NOT ( review or systematic review ) | 629 |
| S6 | Limit to articles in English | 622 |
| S7 | Limit to articles published from January 2015 to present | 434 |

## PsycINFO

Search conducted on March 31, 2023

| **Query** | **Search String** | **Results retrieved** |
| --- | --- | --- |
| 1 | "digital health".mp. or "digital health technology".mp. or exp Mobile Health/ or "mobile health".mp. or "Telehealth".mp. or "telemedicine".mp. or exp Telemedicine/ or "digital care programs".mp. or "digital care programmes".mp. or "health technologies".mp. or "eHealth".mp. or "mHealth".mp. or "digital healthcare".mp. or "wearable medical device".mp. or "mobile medical applications".mp. or "mobile medical software".mp. or "mobile health applications".mp. | 21545 |
| 2 | (exp Wearable Devices/ or "wearable devices".mp. or "wearables".mp. or "wearable technology".mp. or "deep learning".mp. or "machine learning".mp. or exp Machine Learning/ or "artificial intelligence".mp. or exp Artificial Intelligence/ or "human-centered ai".mp. or "human-centered computing".mp. or exp Big Data/ or "big data".mp.) and (exp Health/ or health.mp.) | 3770 |
| 3 | 1 or 2 | 24814 |
| 4 | "implementation".mp. or "translation".mp. or "translational".mp. or "dissemination".mp. or "evaluation".mp. | 482650 |
| 5 | "long-term illness".mp. or "long-term disease".mp. or "long-term health condition".mp. or "chronic illness".mp. or exp Chronic Illness/ or "chronic disease".mp. or "chronic condition".mp. or "chronic disorder".mp. or "chronic health".mp. or "life-long disease".mp. or "life-long condition".mp. or "life-long illness".mp. | 64003 |
| 6 | 3 and 4 and 5 | 279 |
| 7 | 6 not ( review or "systematic review").mp. | 232 |
| 8 | Limit to articles on humans | 225 |
| 9 | Limit to articles in English | 223 |
| 10 | Limit to articles published from January 2015 to present | 117 |

## IEEE Xplore

Search conducted on March 31, 2023

| **Query** | **Search String** | **Results retrieved** |
| --- | --- | --- |
| 1 | ((All Metadata:"Digital health" OR All Metadata:"digital health technology" OR All Metadata:"Mobile health" OR All Metadata:"telehealth" OR All Metadata:"telemedicine" OR All Metadata:"digital care programs" OR All Metadata:"digital care programmes" OR All Metadata:"health technologies" OR All Metadata:"eHealth" OR All Metadata:"mHealth"))) OR ((All Metadata:"digital healthcare" OR All Metadata:"wearable medical device" OR All Metadata:"mobile medical applications" OR All Metadata:"mobile medical software" OR All Metadata:"mobile health application"))) OR ((All Metadata:"health") AND (All Metadata:"wearable devices" OR All Metadata:"wearables" OR All Metadata:"wearable technology" OR All Metadata:"deep learning" OR All Metadata:"machine learning" OR All Metadata:"artificial intelligence" OR All Metadata:"human-centered ai" OR All Metadata:"human-centered computing" OR All Metadata:"big data")) | 41,973 |
| 2 | ("All Metadata":"implementation" OR "All Metadata":"translation" OR "All Metadata":"translational" OR "All Metadata":"dissemination" OR "All Metadata":"evaluation") | 693,325 |
| 3 | ("All Metadata":"Long-term illness" OR "All Metadata":"Long-term disease" OR "All Metadata":"Long-term health condition" OR "All Metadata":"Chronic illness" OR "All Metadata":"Chronic disease" OR "All Metadata":"Chronic condition" OR "All Metadata":"Chronic disorder" OR "All Metadata":"Chronic health" OR "All Metadata":"Life-long disease" OR "All Metadata":"Life-long condition" OR "All Metadata":"Life-long illness") | 1561 |
| 4 | 1 AND 2 AND 3 | 99 |
| 5 | 4 NOT ("All Metadata":"review" OR "All Metadata":"systematic review") | 93 |
| 6 | Limit to articles published from January 2015 to present | 65 |

# S3. Data extraction

| **Data point** | **Description of data extraction** | **Data type** |
| --- | --- | --- |
| Publication year | Publication year of included study | Numerical |
| Authors | Last name of first author | Text |
| Country of study | Specify all countries where the study was conducted. When no country is specifically stated, specify the country of the first author | Text |
| Type of study | Specify the study design, e.g. randomized controlled trial, mixed-method, qualitative description, etc. | Summary text (as some papers may not  explicitly state type of study) |
| Characteristics of the digital health innovation | Specify the type of digital health innovation, target audience, type of chronic disease, sample size, and study duration (start of recruitment to end of data collection) | Summary text |
| Definitions of digital health used by study authors | Specify the definition used | Text |
| Implementation frameworks used | Specify the implementation framework and/or underpinning theory as stated in the article | Summary text |
| Implementation strategies | Specify the implementation strategies used | Summary text |
| Outcome measures used to evaluate implementation | Specify the outcomes as stated in the article and how they have been measured, e.g. which validated instrument was used, data source, etc. | Summary text |
| Key barriers and enablers for successful implementation | Specify the factors as stated in the article that hinder and/or support the implementation process | Summary text |
| Lessons learned | Specify the lessons learned about implementation as stated in the article | Summary text |
| Recommendations shared by the respective study authors | Specify the recommendations provided and suggestions for future research as stated in the article | Summary text |

# S4. List of studies in scoping review

**List of included reports (n=252)**

1. Adeagbo O, Herbst C, Blandford A, McKendry R, Estcourt C, Seeley J, et al. Exploring People's Candidacy for Mobile Health-Supported HIV Testing and Care Services in Rural KwaZulu-Natal, South Africa: Qualitative Study. Journal of Medical Internet Research. 2019;21(11).

2. Ahmed S, Ernst P, Bartlett SJ, Valois MF, Zaihra T, Paré G, et al. The Effectiveness of Web-Based Asthma Self-Management System, My Asthma Portal (MAP): A Pilot Randomized Controlled Trial. J Med Internet Res. 2016;18(12):e313.

3. Ahmedani BK, Crotty N, Abdulhak MM, Ondersma SJ. Pilot feasibility study of a brief, tailored mobile health intervention for depression among patients with chronic pain. Behavioral Medicine. 2015;41(1):25-32.

4. Ahonle ZJ, Kreider CM, Hale-Gallardo J, Castaneda G, Findley K, Ottomanelli L, et al. Implementation and use of video tele-technologies in delivery of individualized community-based vocational rehabilitation services to rural veterans. Journal of Vocational Rehabilitation. 2021;55(2):227-33.

5. Ajayi TA, Salongo L, Zang Y, Wineinger N, Steinhubl S. Mobile Health-Collected Biophysical Markers in Children with Serious Illness-Related Pain. Journal of Palliative Medicine. 2021;24(4):580-8.

6. Al Rajeh A, Steiner MC, Aldabayan Y, Aldhahir A, Pickett E, Quaderi S, et al. Use, utility and methods of telehealth for patients with COPD in England and Wales: a healthcare provider survey. BMJ Open Respir Res. 2019;6(1):e000345.

7. Ali HI, Attlee A, Alhebshi S, Elmi F, Al Dhaheri AS, Stojanovska L, et al. Feasibility Study of a Newly Developed Technology-Mediated Lifestyle Intervention for Overweight and Obese Young Adults. Nutrients. 2021;13(8).

8. AlMahadin G, Lotfi A, Zysk E, Siena FL, Carthy MM, Breedon P. Parkinson's disease: current assessment methods and wearable devices for evaluation of movement disorder motor symptoms - a patient and healthcare professional perspective. BMC Neurol. 2020;20(1):419.

9. Alwakeel AJ, Sicondolfo A, Robitaille C, Bourbeau J, Saad N. The Accessibility, Feasibility, and Safety of a Standardized Community-based Tele-Pulmonary Rehab Program for Chronic Obstructive Pulmonary Disease: A 3-Year Real-World Prospective Study. Ann Am Thorac Soc. 2022;19(1):39-47.

10. Alwashmi MF, Fitzpatrick B, Davis E, Farrell J, Gamble JM, Hawboldt J. Features of a mobile health intervention to manage chronic obstructive pulmonary disease: a qualitative study. Ther Adv Respir Dis. 2020;14:1753466620951044.

11. Alwashmi MF, Fitzpatrick B, Davis E, Gamble JM, Farrell J, Hawboldt J. Perceptions of Health Care Providers Regarding a Mobile Health Intervention to Manage Chronic Obstructive Pulmonary Disease: Qualitative Study. JMIR Mhealth Uhealth. 2019;7(6):e13950.

12. Alwashmi MF, Fitzpatrick B, Farrell J, Gamble JM, Davis E, Nguyen HV, et al. Perceptions of Patients Regarding Mobile Health Interventions for the Management of Chronic Obstructive Pulmonary Disease: Mixed Methods Study. JMIR Mhealth Uhealth. 2020;8(7):e17409.

13. Amann J, Fiordelli M, Brach M, Bertschy S, Scheel-Sailer A, Rubinelli S. Co-designing a Self-Management App Prototype to Support People With Spinal Cord Injury in the Prevention of Pressure Injuries: Mixed Methods Study. JMIR Mhealth Uhealth. 2020;8(7):e18018.

14. Ammenwerth E, Woess S, Baumgartner C, Fetz B, van der Heidt A, Kastner P, et al. Evaluation of an Integrated Telemonitoring Surveillance System in Patients with Coronary Heart Disease. Methods Inf Med. 2015;54(5):388-97.

15. Amorim AB, Pappas E, Simic M, Ferreira ML, Jennings M, Tiedemann A, et al. Integrating Mobile-health, health coaching, and physical activity to reduce the burden of chronic low back pain trial (IMPACT): a pilot randomised controlled trial. BMC Musculoskelet Disord. 2019;20(1):71.

16. Amparo F, Dana R. Web-based longitudinal remote assessment of dry eye symptoms. Ocul Surf. 2018;16(2):249-53.

17. Andersen TO, Langstrup H, Lomborg S. Experiences With Wearable Activity Data During Self-Care by Chronic Heart Patients: Qualitative Study. J Med Internet Res. 2020;22(7):e15873.

18. Anderson LM, Leonard S, Jonassaint J, Lunyera J, Bonner M, Shah N. Mobile health intervention for youth with sickle cell disease: Impact on adherence, disease knowledge, and quality of life. Pediatr Blood Cancer. 2018;65(8):e27081.

19. Andrén P, Aspvall K, Fernández de la Cruz L, Wiktor P, Romano S, Andersson E, et al. Therapist-guided and parent-guided internet-delivered behaviour therapy for paediatric Tourette's disorder: a pilot randomised controlled trial with long-term follow-up. BMJ Open. 2019;9(2):e024685.

20. Anglada-Martínez H, Martin-Conde M, Rovira-Illamola M, Sotoca-Momblona JM, Sequeira E, Aragunde V, et al. Feasibility and Preliminary Outcomes of a Web and Smartphone-Based Medication Self-Management Platform for Chronically Ill Patients. J Med Syst. 2016;40(4):99.

21. Ariens LF, Schussler-Raymakers FM, Frima C, Flinterman A, Hamminga E, Arents BW, et al. Barriers and Facilitators to eHealth Use in Daily Practice: Perspectives of Patients and Professionals in Dermatology. J Med Internet Res. 2017;19(9):e300.

22. Ayatollahi H, Hasannezhad M, Fard HS, Haghighi MK. Type 1 diabetes self-management: developing a web-based telemedicine application. Health Inf Manag. 2016;45(1):16-26.

23. Banerjee A, Ramanujan RA, Agnihothri S, editors. Mobile Health Monitoring: Development and Implementation of an App in a Diabetes and Hypertension Clinic. 2016 49th Hawaii International Conference on System Sciences (HICSS); 2016 5-8 Jan. 2016.

24. Barakat-Johnson M, Kita B, Jones A, Burger M, Airey D, Stephenson J, et al. The viability and acceptability of a Virtual Wound Care Command Centre in Australia. Int Wound J. 2022;19(7):1769-85.

25. Barenfeld E, Fuller JM, Wallström S, Fors A, Ali L, Ekman I. Meaningful use of a digital platform and structured telephone support to facilitate remote person-centred care - a mixed-method study on patient perspectives. BMC Health Serv Res. 2022;22(1):442.

26. Bauer MS, Krawczyk L, Tuozzo K, Frigand C, Holmes S, Miller CJ, et al. Implementing and Sustaining Team-Based Telecare for Bipolar Disorder: Lessons Learned from a Model-Guided, Mixed Methods Analysis. Telemed J E Health. 2018;24(1):45-53.

27. Bello AK, Molzahn AE, Girard LP, Osman MA, Okpechi IG, Glassford J, et al. Patient and provider perspectives on the design and implementation of an electronic consultation system for kidney care delivery in Canada: a focus group study. BMJ Open. 2017;7(3):e014784.

28. Ben-Zeev D, Brian RM, Jonathan G, Razzano L, Pashka N, Carpenter-Song E, et al. Mobile Health (mHealth) Versus Clinic-Based Group Intervention for People With Serious Mental Illness: A Randomized Controlled Trial. Psychiatr Serv. 2018;69(9):978-85.

29. Bender C, Hangaard S, Kronborg T, Hejlesen OK, Secher PH. Preliminary Qualitative Evaluation of Patient-Related Perspectives Related to the Implementation of a Predictive Algorithm in a Telehealth System for COPD. Stud Health Technol Inform. 2021;281:545-9.

30. Bentley CL, Powell L, Potter S, Parker J, Mountain GA, Bartlett YK, et al. The Use of a Smartphone App and an Activity Tracker to Promote Physical Activity in the Management of Chronic Obstructive Pulmonary Disease: Randomized Controlled Feasibility Study. JMIR Mhealth Uhealth. 2020;8(6):e16203.

31. Benzo RP, Kramer KM, Hoult JP, Anderson PM, Begue IM, Seifert SJ. Development and Feasibility of a Home Pulmonary Rehabilitation Program With Health Coaching. Respir Care. 2018;63(2):131-40.

32. Bhatia A, Kara J, Janmohamed T, Prabhu A, Lebovic G, Katz J, et al. User Engagement and Clinical Impact of the Manage My Pain App in Patients With Chronic Pain: A Real-World, Multi-site Trial. JMIR Mhealth Uhealth. 2021;9(3):e26528.

33. Bonacini M, Kim Y, Pitney C, McKoin L, Tran M, Landis C. Wirelessly Observed Therapy to Optimize Adherence and Target Interventions for Oral Hepatitis C Treatment: Observational Pilot Study. J Med Internet Res. 2020;22(2):e15532.

34. Botros A, Schütz N, Camenzind M, Urwyler P, Bolliger D, Vanbellingen T, et al. Long-Term Home-Monitoring Sensor Technology in Patients with Parkinson's Disease-Acceptance and Adherence. Sensors (Basel). 2019;19(23).

35. Brouwers RWM, Kemps HMC, Herkert C, Peek N, Kraal JJ. A 12-week cardiac telerehabilitation programme does not prevent relapse of physical activity levels: long-term results of the FIT@Home trial. Eur J Prev Cardiol. 2022;29(7):e255-e7.

36. Bruggeman-Everts FZ, Wolvers MDJ, van de Schoot R, Vollenbroek-Hutten MMR, Van der Lee ML. Effectiveness of Two Web-Based Interventions for Chronic Cancer-Related Fatigue Compared to an Active Control Condition: Results of the "Fitter na kanker" Randomized Controlled Trial. J Med Internet Res. 2017;19(10):e336.

37. Carlsen K, Jakobsen C, Houen G, Kallemose T, Paerregaard A, Riis LB, et al. Self-managed eHealth Disease Monitoring in Children and Adolescents with Inflammatory Bowel Disease: A Randomized Controlled Trial. Inflamm Bowel Dis. 2017;23(3):357-65.

38. Cella M, Okruszek Ł, Lawrence M, Zarlenga V, He Z, Wykes T. Using wearable technology to detect the autonomic signature of illness severity in schizophrenia. Schizophr Res. 2018;195:537-42.

39. Chan C, Inskip JA, Kirkham AR, Ansermino JM, Dumont G, Li LC, et al. A smartphone oximeter with a fingertip probe for use during exercise training: usability, validity and reliability in individuals with chronic lung disease and healthy controls. Physiotherapy. 2019;105(3):297-306.

40. Choi YH, Park HK, Paik NJ. A Telerehabilitation Approach for Chronic Aphasia Following Stroke. Telemed J E Health. 2016;22(5):434-40.

41. Cingi C, Yorgancioglu A, Cingi CC, Oguzulgen K, Muluk NB, Ulusoy S, et al. The "physician on call patient engagement trial" (POPET): measuring the impact of a mobile patient engagement application on health outcomes and quality of life in allergic rhinitis and asthma patients. Int Forum Allergy Rhinol. 2015;5(6):487-97.

42. Claes J, Cornelissen V, McDermott C, Moyna N, Pattyn N, Cornelis N, et al. Feasibility, Acceptability, and Clinical Effectiveness of a Technology-Enabled Cardiac Rehabilitation Platform (Physical Activity Toward Health-I): Randomized Controlled Trial. J Med Internet Res. 2020;22(2):e14221.

43. Cohn WF, Canan CE, Knight S, Waldman AL, Dillingham R, Ingersoll K, et al. An Implementation Strategy to Expand Mobile Health Use in HIV Care Settings: Rapid Evaluation Study Using the Consolidated Framework for Implementation Research. JMIR Mhealth Uhealth. 2021;9(4):e19163.

44. Cooke M, Richards J, Tjondronegoro D, Raj Chakraborty P, Jauncey-Cooke J, Andresen E, et al. myPainPal: Co-creation of a mHealth app for the management of chronic pain in young people. Inform Health Soc Care. 2021;46(3):291-305.

45. Costa Stutzel M, Filippo MP, Sztajnberg A, da Costa R, Brites ADS, da Motta LB, et al. Multi-part quality evaluation of a customized mobile application for monitoring elderly patients with functional loss and helping caregivers. BMC Med Inform Decis Mak. 2019;19(1):140.

46. Crawford MR, Luik AI, Espie CA, Taylor HL, Burgess HJ, Jones AL, et al. Digital Cognitive Behavioral Therapy for Insomnia in Women With Chronic Migraines. Headache. 2020;60(5):902-15.

47. D'Haeseleer M, Eelen P, Sadeghi N, D'Hooghe MB, Van Schependom J, Nagels G. Feasibility of Real Time Internet-Based Teleconsultation in Patients With Multiple Sclerosis: Interventional Pilot Study. J Med Internet Res. 2020;22(8):e18178.

48. Damhus CS, Emme C, Hansen H. Barriers and enablers of COPD telerehabilitation - a frontline staff perspective. Int J Chron Obstruct Pulmon Dis. 2018;13:2473-82.

49. Davis LE, Harnar J, LaChey-Barbee LA, Pirio Richardson S, Fraser A, King MK. Using Teleneurology to Deliver Chronic Neurologic Care to Rural Veterans: Analysis of the First 1,100 Patient Visits. Telemed J E Health. 2019;25(4):274-8.

50. de Batlle J, Massip M, Vargiu E, Nadal N, Fuentes A, Ortega Bravo M, et al. Implementing Mobile Health-Enabled Integrated Care for Complex Chronic Patients: Patients and Professionals' Acceptability Study. JMIR Mhealth Uhealth. 2020;8(11):e22136.

51. de Jong M, van der Meulen-de Jong A, Romberg-Camps M, Degens J, Becx M, Markus T, et al. Development and Feasibility Study of a Telemedicine Tool for All Patients with IBD: MyIBDcoach. Inflamm Bowel Dis. 2017;23(4):485-93.

52. Deng N, Chen J, Liu Y, Wei S, Sheng L, Lu R, et al. Using Mobile Health Technology to Deliver a Community-Based Closed-Loop Management System for Chronic Obstructive Pulmonary Disease Patients in Remote Areas of China: Development and Prospective Observational Study. JMIR Mhealth Uhealth. 2020;8(11):e15978.

53. Dhakal R, Baniya M, Solomon RM, Rana C, Ghimire P, Hariharan R, et al. TEleRehabilitation Nepal (TERN) for People With Spinal Cord Injury and Acquired Brain Injury: A Feasibility Study. Rehabilitation Process & Outcome. 2022:1-13.

54. Diez-Canseco F, Toyama M, Ipince A, Perez-Leon S, Cavero V, Araya R, et al. Integration of a Technology-Based Mental Health Screening Program Into Routine Practices of Primary Health Care Services in Peru (The Allillanchu Project): Development and Implementation. J Med Internet Res. 2018;20(3):e100.

55. Dodakian L, McKenzie AL, Le V, See J, Pearson-Fuhrhop K, Burke Quinlan E, et al. A Home-Based Telerehabilitation Program for Patients With Stroke. Neurorehabil Neural Repair. 2017;31(10-11):923-33.

56. Donald M, Beanlands H, Straus S, Smekal M, Gil S, Elliott MJ, et al. An eHealth self-management intervention for adults with chronic kidney disease, My Kidneys My Health: a mixed-methods study. CMAJ Open. 2022;10(3):E746-e54.

57. Donesky D, Selman L, McDermott K, Citron T, Howie-Esquivel J. Evaluation of the Feasibility of a Home-Based TeleYoga Intervention in Participants with Both Chronic Obstructive Pulmonary Disease and Heart Failure. J Altern Complement Med. 2017;23(9):713-21.

58. Doyle J, Murphy E, Gavin S, Pascale A, Deparis S, Tommasi P, et al. A Digital Platform to Support Self-management of Multiple Chronic Conditions (ProACT): Findings in Relation to Engagement During a One-Year Proof-of-Concept Trial. J Med Internet Res. 2021;23(12):e22672.

59. Doyle N, Murphy M, Brennan L, Waugh A, McCann M, Mellotte G. The "Mikidney" smartphone app pilot study: Empowering patients with Chronic Kidney Disease. J Ren Care. 2019;45(3):133-40.

60. Duan H, Wang Z, Ji Y, Ma L, Liu F, Chi M, et al. Using Goal-Directed Design to Create a Mobile Health App to Improve Patient Compliance With Hypertension Self-Management: Development and Deployment. JMIR Mhealth Uhealth. 2020;8(2):e14466.

61. Easton K, Potter S, Bec R, Bennion M, Christensen H, Grindell C, et al. A Virtual Agent to Support Individuals Living With Physical and Mental Comorbidities: Co-Design and Acceptability Testing. J Med Internet Res. 2019;21(5):e12996.

62. Eisner E, Drake RJ, Berry N, Barrowclough C, Emsley R, Machin M, et al. Development and Long-Term Acceptability of ExPRESS, a Mobile Phone App to Monitor Basic Symptoms and Early Signs of Psychosis Relapse. JMIR Mhealth Uhealth. 2019;7(3):e11568.

63. Farmer A, Williams V, Velardo C, Shah SA, Yu LM, Rutter H, et al. Self-Management Support Using a Digital Health System Compared With Usual Care for Chronic Obstructive Pulmonary Disease: Randomized Controlled Trial. J Med Internet Res. 2017;19(5):e144.

64. Fernon A, Nguyen A, Baysari M, Day R. A User-Centred Approach to Designing an eTool for Gout Management. Studies in Health Technology & Informatics. 2016;227:28-33.

65. Fisher JM, Hammerla NY, Rochester L, Andras P, Walker RW. Body-Worn Sensors in Parkinson's Disease: Evaluating Their Acceptability to Patients. Telemed J E Health. 2016;22(1):63-9.

66. Floch J, Vilarinho T, Zettl A, Ibanez-Sanchez G, Calvo-Lerma J, Stav E, et al. Users' Experiences of a Mobile Health Self-Management Approach for the Treatment of Cystic Fibrosis: Mixed Methods Study. JMIR Mhealth Uhealth. 2020;8(7):e15896.

67. Flujas-Contreras JM, Ruiz-Castañeda D, Gómez I. Promoting Emotional Well-being in Hospitalized Children and Adolescents With Virtual Reality: Usability and Acceptability of a Randomized Controlled Trial. Comput Inform Nurs. 2020;38(2):99-107.

68. Forbes G, Newton S, Cantalapiedra Calvete C, Birch J, Dodds J, Steed L, et al. MEMPHIS: a smartphone app using psychological approaches for women with chronic pelvic pain presenting to gynaecology clinics: a randomised feasibility trial. BMJ Open. 2020;10(3):e030164.

69. Ford AR, Gibbons CM, Torres J, Kornmehl HA, Singh S, Young PM, et al. Access to Dermatological Care with an Innovative Online Model for Psoriasis Management: Results from a Randomized Controlled Trial. Telemed J E Health. 2019;25(7):619-27.

70. Fortuna KL, Myers AL, Ferron J, Kadakia A, Bianco C, Bruce ML, et al. Assessing a digital peer support self-management intervention for adults with serious mental illness: feasibility, acceptability, and preliminary effectiveness. Journal of Mental Health. 2022;31(6):833-41.

71. Frederix I, Hansen D, Coninx K, Vandervoort P, Vandijck D, Hens N, et al. Medium-Term Effectiveness of a Comprehensive Internet-Based and Patient-Specific Telerehabilitation Program With Text Messaging Support for Cardiac Patients: Randomized Controlled Trial. J Med Internet Res. 2015;17(7):e185.

72. Fritz JM, Minick KI, Brennan GP, McGee T, Lane E, Skolasky RL, et al. Outcomes of Telehealth Physical Therapy Provided Using Real-Time, Videoconferencing for Patients With Chronic Low Back Pain: A Longitudinal Observational Study. Arch Phys Med Rehabil. 2022;103(10):1924-34.

73. Gabbard J, McLouth CJ, Brenes G, Claudel S, Ongchuan S, Burkart J, et al. Rapid Electronic Capturing of Patient-Reported Outcome Measures in Older Adults With End-Stage Renal Disease: A Feasibility Study. Am J Hosp Palliat Care. 2021;38(5):432-40.

74. Geirhos A, Domhardt M, Lunkenheimer F, Temming S, Holl RW, Minden K, et al. Feasibility and potential efficacy of a guided internet- and mobile-based CBT for adolescents and young adults with chronic medical conditions and comorbid depression or anxiety symptoms (youthCOACH(CD)): a randomized controlled pilot trial. BMC Pediatr. 2022;22(1):69.

75. Glattacker M, Boeker M, Anger R, Reichenbach F, Tassoni A, Bredenkamp R, et al. Evaluation of a Mobile Phone App for Patients With Pollen-Related Allergic Rhinitis: Prospective Longitudinal Field Study. JMIR Mhealth Uhealth. 2020;8(4):e15514.

76. Gómez-Restrepo C, Cepeda M, Torrey W, Castro S, Uribe-Restrepo JM, Suárez-Obando F, et al. The DIADA project: A technology-based model of care for depression and risky alcohol use in primary care centres in Colombia. Rev Colomb Psiquiatr (Engl Ed). 2021;50 Suppl 1(Suppl 1):4-12.

77. Gordon HS, Solanki P, Bokhour BG, Gopal RK. "I'm Not Feeling Like I'm Part of the Conversation" Patients' Perspectives on Communicating in Clinical Video Telehealth Visits. J Gen Intern Med. 2020;35(6):1751-8.

78. Guo X, Gu X, Jiang J, Li H, Duan R, Zhang Y, et al. A Hospital-Community-Family-Based Telehealth Program for Patients With Chronic Heart Failure: Single-Arm, Prospective Feasibility Study. JMIR Mhealth Uhealth. 2019;7(12):e13229.

79. Guo X, Yang Y, Takiff HE, Zhu M, Ma J, Zhong T, et al. A Comprehensive App That Improves Tuberculosis Treatment Management Through Video-Observed Therapy: Usability Study. JMIR Mhealth Uhealth. 2020;8(7):e17658.

80. Hale TM, Jethwani K, Kandola MS, Saldana F, Kvedar JC. A Remote Medication Monitoring System for Chronic Heart Failure Patients to Reduce Readmissions: A Two-Arm Randomized Pilot Study. Journal of Medical Internet Research. 2016;18(4):e91.

81. Halterman JS, Fagnano M, Tajon RS, Tremblay P, Wang H, Butz A, et al. Effect of the School-Based Telemedicine Enhanced Asthma Management (SB-TEAM) Program on Asthma Morbidity: A Randomized Clinical Trial. JAMA Pediatrics. 2018;172(3):e174938.

82. Hanley J, Fairbrother P, McCloughan L, Pagliari C, Paterson M, Pinnock H, et al. Qualitative study of telemonitoring of blood glucose and blood pressure in type 2 diabetes. BMJ Open. 2015;5(12):e008896.

83. Hauser-Ulrich S, Künzli H, Meier-Peterhans D, Kowatsch T. A Smartphone-Based Health Care Chatbot to Promote Self-Management of Chronic Pain (SELMA): Pilot Randomized Controlled Trial. JMIR Mhealth Uhealth. 2020;8(4):e15806.

84. Haynes SC, Kim KK. A mobile system for the improvement of heart failure management: Evaluation of a prototype. AMIA Annu Symp Proc. 2017;2017:839-48.

85. Herrmann S, Power B, Rashidi A, Cypher M, Mastaglia F, Grace A, et al. Supporting Patient-Clinician Interaction in Chronic HIV Care: Design and Development of a Patient-Reported Outcomes Software Application. J Med Internet Res. 2021;23(7):e27861.

86. Hicks TAB, Thomas SP, Wilson SM, Calhoun PS, Kuhn ER, Beckham JC. A Preliminary Investigation of a Relapse Prevention Mobile Application to Maintain Smoking Abstinence Among Individuals With Posttraumatic Stress Disorder. J Dual Diagn. 2017;13(1):15-20.

87. Ho K, Newton L, Boothe A, Novak-Lauscher H. mobile Digital Access to a Web-enhanced Network (mDAWN): Assessing the Feasibility of Mobile Health Tools for Self-Management of Type-2 Diabetes. AMIA Annu Symp Proc. 2015;2015:621-9.

88. Huygens MWJ, Voogdt-Pruis HR, Wouters M, Meurs MM, Lettow Bv, Kleijweg C, et al. The Uptake and Use of Telemonitoring in Chronic Care Between 2014 and 2019: Nationwide Survey Among Patients and Health Care Professionals in the Netherlands. Journal of Medical Internet Research. 2021;23(5):N.PAG-N.PAG.

89. Imtiaz R, Atkinson K, Guerinet J, Wilson K, Leidecker J, Zimmerman D. A Pilot Study of OkKidney, a Phosphate Counting Application in Patients on Peritoneal Dialysis. Perit Dial Int. 2017;37(6):613-8.

90. Infarinato F, Jansen-Kosterink S, Romano P, van Velsen L, Op den Akker H, Rizza F, et al. Acceptance and Potential Impact of the eWALL Platform for Health Monitoring and Promotion in Persons with a Chronic Disease or Age-Related Impairment. Int J Environ Res Public Health. 2020;17(21).

91. Jaana M, Sherrard H, Paré G. A prospective evaluation of telemonitoring use by seniors with chronic heart failure: Adoption, self-care, and empowerment. Health Informatics J. 2019;25(4):1800-14.

92. Jain YS, Garg A, Jhamb DK, Jain P, Karar A. Preparing India to Leverage Power of Mobile Technology: Development of a Bilingual Mobile Health Tool for Heart Patients. Cardiovasc Hematol Agents Med Chem. 2019;17(2):125-34.

93. Jakobsen AS, Laursen LC, Rydahl-Hansen S, Østergaard B, Gerds TA, Emme C, et al. Home-based telehealth hospitalization for exacerbation of chronic obstructive pulmonary disease: findings from "the virtual hospital" trial. Telemed J E Health. 2015;21(5):364-73.

94. Jakubowski KP, Jhamb M, Yabes J, Gujral S, Oberlin LE, Bender FH, et al. Technology-assisted cognitive-behavioral therapy intervention for end-stage renal disease. Translational Behavioral Medicine. 2020;10(3):657-63.

95. Jansen-Kosterink S, Dekker-van Weering M, van Velsen L. Patient acceptance of a telemedicine service for rehabilitation care: A focus group study. International Journal of Medical Informatics. 2019;125:22-9.

96. Jiang Y, Sun P, Chen Z, Guo J, Wang S, Liu F, et al. Patients' and healthcare providers' perceptions and experiences of telehealth use and online health information use in chronic disease management for older patients with chronic obstructive pulmonary disease: a qualitative study. BMC Geriatrics. 2022;22(1):1-16.

97. Jiménez-Reguera B, Maroto López E, Fitch S, Juarros L, Sánchez Cortés M, Rodríguez Hermosa JL, et al. Development and Preliminary Evaluation of the Effects of an mHealth Web-Based Platform (HappyAir) on Adherence to a Maintenance Program After Pulmonary Rehabilitation in Patients With Chronic Obstructive Pulmonary Disease: Randomized Controlled Trial. JMIR Mhealth Uhealth. 2020;8(7):e18465.

98. Jindal D, Roy A, Ajay VS, Yadav SK, Prabhakaran D, Tandon N. Strategies for Stakeholder Engagement and Uptake of New Intervention: Experience From State-Wide Implementation of mHealth Technology for NCD Care in Tripura, India. Glob Heart. 2019;14(2):165-72.

99. Johnson EE, MacGeorge C, King KL, Andrews AL, Teufel RJ, 2nd, Kruis R, et al. Facilitators and Barriers to Implementation of School-Based Telehealth Asthma Care: Program Champion Perspectives. Acad Pediatr. 2021;21(7):1262-72.

100. Jonassaint CR, Kang C, Prussien KV, Yarboi J, Sanger MS, Wilson JD, et al. Feasibility of implementing mobile technology-delivered mental health treatment in routine adult sickle cell disease care. Transl Behav Med. 2020;10(1):58-67.

101. Jonathan G, Carpenter-Song EA, Brian RM, Ben-Zeev D. Life with FOCUS: A qualitative evaluation of the impact of a smartphone intervention on people with serious mental illness. Psychiatric Rehabilitation Journal. 2019;42(2):182-9.

102. Jongen PJ, Veen Gt, Lemmens W, Donders R, Noort Ev, Zeinstra E, et al. The Interactive Web-Based Program MSmonitor for Self-Management and Multidisciplinary Care in Persons With Multiple Sclerosis: Quasi-Experimental Study of Short-Term Effects on Patient Empowerment. Journal of Medical Internet Research. 2020;22(3):e14297.

103. Jordan DN, Jessen CM, Ferucci ED. Views of Patients and Providers on the Use of Telemedicine for Chronic Disease Specialty Care in the Alaska Native Population. Telemed J E Health. 2021;27(1):82-9.

104. Kadiri SB, Kerr AP, Oswald NK, Budacan AM, Flanagan S, Golby C, et al. Fit 4 surgery, a bespoke app with biofeedback delivers rehabilitation at home before and after elective lung resection. J Cardiothorac Surg. 2019;14(1):132.

105. Kamei T, Yamamoto Y, Kanamori T, Nakayama Y, Porter SE. Detection of early-stage changes in people with chronic diseases: A telehome monitoring-based telenursing feasibility study. Nursing & Health Sciences. 2018;20(3):313-22.

106. Kao DP, Lindenfeld J, Macaulay D, Birnbaum HG, Jarvis JL, Desai US, et al. Impact of a Telehealth and Care Management Program on All-Cause Mortality and Healthcare Utilization in Patients with Heart Failure. Telemed J E Health. 2016;22(1):2-11.

107. Katz IJ, Pirabhahar S, Williamson P, Raghunath V, Brennan F, O'Sullivan A, et al. iConnect CKD - virtual medical consulting: A web-based chronic kidney disease, hypertension and diabetes integrated care program. Nephrology (Carlton). 2018;23(7):646-52.

108. Kayser MZ, Valtin C, Greer M, Karow B, Fuge J, Gottlieb J. Video Consultation During the COVID-19 Pandemic: A Single Center's Experience with Lung Transplant Recipients. Telemed J E Health. 2021;27(7):807-15.

109. Kazankov K, Novelli S, Chatterjee DA, Phillips A, Balaji A, Raja M, et al. Evaluation of CirrhoCare® - a digital health solution for home management of individuals with cirrhosis. J Hepatol. 2023;78(1):123-32.

110. Kenealy TW, Parsons MJ, Rouse AP, Doughty RN, Sheridan NF, Hindmarsh JK, et al. Telecare for diabetes, CHF or COPD: effect on quality of life, hospital use and costs. A randomised controlled trial and qualitative evaluation. PLoS One. 2015;10(3):e0116188.

111. Kettlewell J, Phillips J, Radford K, dasNair R. Informing evaluation of a smartphone application for people with acquired brain injury: a stakeholder engagement study. BMC Med Inform Decis Mak. 2018;18(1):33.

112. Khan F, Granville N, Malkani R, Chathampally Y. Health-Related Quality of Life Improvements in Systemic Lupus Erythematosus Derived from a Digital Therapeutic Plus Tele-Health Coaching Intervention: Randomized Controlled Pilot Trial. Journal of Medical Internet Research. 2020;22(10).

113. Kim A, Yun SJ, Sung KS, Kim Y, Jo JY, Cho H, et al. Exercise Management Using a Mobile App in Patients With Parkinsonism: Prospective, Open-Label, Single-Arm Pilot Study. JMIR Mhealth Uhealth. 2021;9(8):e27662.

114. Knox L, Dunning M, Davies CA, Mills-Bennet R, Sion TW, Phipps K, et al. Safety, feasibility, and effectiveness of virtual pulmonary rehabilitation in the real world. Int J Chron Obstruct Pulmon Dis. 2019;14:775-80.

115. Kock AK, Kaya RS, Müller C, Andersen B, Langer T, Ingenerf J. Design, implementation, and evaluation of a mobile application for patient empowerment and management of long-term follow-up after childhood cancer. Klin Padiatr. 2015;227(3):166-70.

116. Kondylakis H, Bucur A, Crico C, Dong F, Graf N, Hoffman S, et al. Patient empowerment for cancer patients through a novel ICT infrastructure. Journal of Biomedical Informatics. 2020;101.

117. Kooij L, Vos PJE, Dijkstra A, van Harten WH. Effectiveness of a Mobile Health and Self-Management App for High-Risk Patients With Chronic Obstructive Pulmonary Disease in Daily Clinical Practice: Mixed Methods Evaluation Study. JMIR Mhealth Uhealth. 2021;9(2):e21977.

118. Kouri A, Yamada J, Sale JEM, Straus SE, Gupta S. Primary Care Pre-Visit Electronic Patient Questionnaire for Asthma: Uptake Analysis and Predictor Modeling. J Med Internet Res. 2020;22(9):e19358.

119. Krkoska P, Vlazna D, Sladeckova M, Minarikova J, Barusova T, Batalik L, et al. Adherence and Effect of Home-Based Rehabilitation with Telemonitoring Support in Patients with Chronic Non-Specific Low Back Pain: A Pilot Study. Int J Environ Res Public Health. 2023;20(2).

120. Kumar A, Lall N, Pathak A, Joshi D, Mishra VN, Chaurasia RN, et al. A questionnaire-based survey of acceptability and satisfaction of virtual neurology clinic during COVID-19 lockdown: a preliminary study. Acta Neurol Belg. 2022;122(5):1297-304.

121. Kvedarienė V, Biliute G, Didziokaitė G, Kavaliukaite L, Savonyte A, Rudzikaite-Fergize G, et al. Mobile health app for monitoring allergic rhinitis and asthma in real life in Lithuanian MASK-air users. Clinical and Translational Allergy. 2022;12(9).

122. Kwok JYY, Lee JJ, Choi EPH, Chau PH, Auyeung M. Stay mindfully active during the coronavirus pandemic: a feasibility study of mHealth-delivered mindfulness yoga program for people with Parkinson's disease. BMC Complement Med Ther. 2022;22(1):37.

123. Lalloo C, Hundert A, Harris L, Pham Q, Campbell F, Chorney J, et al. Capturing Daily Disease Experiences of Adolescents With Chronic Pain: mHealth-Mediated Symptom Tracking. JMIR Mhealth Uhealth. 2019;7(1):e11838.

124. Lalloo C, Nishat F, Zempsky W, Bakshi N, Badawy S, Ko YJ, et al. Characterizing User Engagement With a Digital Intervention for Pain Self-management Among Youth With Sickle Cell Disease and Their Caregivers: Subanalysis of a Randomized Controlled Trial. J Med Internet Res. 2022;24(8):e40096.

125. Lambert J, Taylor A, Streeter A, Greaves C, Ingram WM, Dean S, et al. A process evaluation, with mediation analysis, of a web-based intervention to augment primary care exercise referral schemes: the e-coachER randomised controlled trial. Int J Behav Nutr Phys Act. 2022;19(1):128.

126. Lapão LV, Peyroteo M, Maia M, Seixas J, Gregório J, Mira da Silva M, et al. Implementation of Digital Monitoring Services During the COVID-19 Pandemic for Patients With Chronic Diseases: Design Science Approach. J Med Internet Res. 2021;23(8):e24181.

127. Le Goff-Pronost M, Mourgeon B, Blanchère JP, Teot L, Benateau H, Dompmartin A. Real-World Clinical Evaluation and Costs of Telemedicine for Chronic Wound Management. Int J Technol Assess Health Care. 2018;34(6):567-75.

128. Lee JY, Chan CKY, Chua SS, Paraidathathu T, Lee KK, Tan CSS, et al. Using telemedicine to support care for people with type 2 diabetes mellitus: a qualitative analysis of patients' perspectives. BMJ Open. 2019;9(10):e026575.

129. Lehmann J, Buhl P, Giesinger JM, Wintner LM, Sztankay M, Neppl L, et al. Using the Computer-based Health Evaluation System (CHES) to Support Self-management of Symptoms and Functional Health: Evaluation of Hematological Patient Use of a Web-Based Patient Portal. J Med Internet Res. 2021;23(6):e26022.

130. Lewis A, Knight E, Bland M, Middleton J, Mitchell E, McCrum K, et al. Feasibility of an online platform delivery of pulmonary rehabilitation for individuals with chronic respiratory disease. BMJ Open Respir Res. 2021;8(1).

131. Li J, Varnfield M, Jayasena R, Celler B. Home telemonitoring for chronic disease management: Perceptions of users and factors influencing adoption. Health Informatics Journal. 2021;27(1):1-17.

132. Li WY, Chiu FC, Zeng JK, Li YW, Huang SH, Yeh HC, et al. Mobile Health App With Social Media to Support Self-Management for Patients With Chronic Kidney Disease: Prospective Randomized Controlled Study. J Med Internet Res. 2020;22(12):e19452.

133. Lin JL, Huber B, Amir O, Gehrmann S, Ramirez KS, Ochoa KM, et al. Barriers and Facilitators to the Implementation of Family-Centered Technology in Complex Care: Feasibility Study. J Med Internet Res. 2022;24(8):e30902.

134. Liu Y, Zupan NJ, Swearingen R, Jacobson N, Carlson JN, Mahoney JE, et al. Identification of barriers, facilitators and system-based implementation strategies to increase teleophthalmology use for diabetic eye screening in a rural US primary care clinic: a qualitative study. BMJ Open. 2019;9(2):e022594.

135. Looman WS, Antolick M, Cady RG, Lunos SA, Garwick AE, Finkelstein SM. Effects of a telehealth care coordination intervention on perceptions of health care by caregivers of children with medical complexity: A randomized controlled trial. Journal of Pediatric Health Care. 2015;29(4):352-63.

136. Lopez JJ, Svetanoff WJ, Rosen JM, Carrasco A, Rentea RM. Leveraging Collaboration in Pediatric Multidisciplinary Colorectal Care Using a Telehealth Platform. Am Surg. 2022;88(9):2320-6.

137. Lundell S, Modig M, Holmner A, Wadell K. Perceptions of home telemonitoring use among patients with chronic obstructive pulmonary disease: Qualitative study. JMIR Mhealth Uhealth. 2020;8(6):e16343.

138. Mammen JR, Schoonmaker JD, Java J, Halterman J, Berliant MN, Crowley A, et al. Going mobile with primary care: smartphone-telemedicine for asthma management in young urban adults (TEAMS). J Asthma. 2022;59(1):132-44.

139. Martínez García MA, Fernández Rosales MS, López Domínguez E, Hernández Velázquez Y, Domínguez Isidro S. Telemonitoring system for patients with chronic kidney disease undergoing peritoneal dialysis: Usability assessment based on a case study. PLoS One. 2018;13(11):e0206600.

140. Martínez P, Guajardo V, Gómez VE, Brandt S, Szabo W, Soto-Brandt G, et al. Technology-Assisted Collaborative Care Program for People with Diabetes and/or High Blood Pressure Attending Primary Health Care: A Feasibility Study. Int J Environ Res Public Health. 2021;18(22).

141. Martinez RN, Hogan TP, Balbale S, Lones K, Goldstein B, Woo C, et al. Sociotechnical Perspective on Implementing Clinical Video Telehealth for Veterans with Spinal Cord Injuries and Disorders. Telemed J E Health. 2017;23(7):567-76.

142. Martinez RN, Hogan TP, Lones K, Balbale S, Scholten J, Bidelspach D, et al. Evaluation and Treatment of Mild Traumatic Brain Injury Through the Implementation of Clinical Video Telehealth: Provider Perspectives From the Veterans Health Administration. Pm r. 2017;9(3):231-40.

143. Mattocks KM, LaChappelle KM, Krein SL, DeBar LL, Martino S, Edmond S, et al. Pre-implementation formative evaluation of cooperative pain education and self-management expanding treatment for real-world access: A pragmatic pain trial. Pain Pract. 2023;23(4):338-48.

144. Maxwell LG, McFarland MS, Baker JW, Cassidy RF. Evaluation of the Impact of a Pharmacist-Led Telehealth Clinic on Diabetes-Related Goals of Therapy in a Veteran Population. Pharmacotherapy. 2016;36(3):348-56.

145. Monteiro-Guerra F, Signorelli GR, Rivera-Romero O, Dorronzoro-Zubiete E, Caulfield B. Breast Cancer Survivors' Perspectives on Motivational and Personalization Strategies in Mobile App-Based Physical Activity Coaching Interventions: Qualitative Study. JMIR Mhealth Uhealth. 2020;8(9):e18867.

146. Morano JP, Clauson K, Zhou Z, Escobar-Viera CG, Lieb S, Chen IK, et al. Attitudes, Beliefs, and Willingness Toward the Use of mHealth Tools for Medication Adherence in the Florida mHealth Adherence Project for People Living With HIV (FL-mAPP): Pilot Questionnaire Study. JMIR Mhealth Uhealth. 2019;7(7):e12900.

147. Morisada MV, Hwang J, Gill AS, Wilson MD, Strong EB, Steele TO. Telemedicine, Patient Satisfaction, and Chronic Rhinosinusitis Care in the Era of COVID-19. Am J Rhinol Allergy. 2021;35(4):494-9.

148. Morita PP, Yeung MS, Ferrone M, Taite AK, Madeley C, Stevens Lavigne A, et al. A Patient-Centered Mobile Health System That Supports Asthma Self-Management (breathe): Design, Development, and Utilization. JMIR Mhealth Uhealth. 2019;7(1):e10956.

149. Mucchi L, Jayousi S, Gant A, Paoletti E, Zoppi P. Tele-Monitoring System for Chronic Diseases Management: Requirements and Architecture. Int J Environ Res Public Health. 2021;18(14):7459.

150. Muroff J, Robinson W, Chassler D, López LM, Gaitan E, Lundgren L, et al. Use of a Smartphone Recovery Tool for Latinos with Co-Occurring Alcohol and Other Drug Disorders and Mental Disorders. J Dual Diagn. 2017;13(4):280-90.

151. Nagel T, Sweet M, Dingwall KM, Puszka S, Hughes JT, Kavanagh DJ, et al. Adapting wellbeing research tools for Aboriginal and Torres Strait Islander people with chronic kidney disease. BMC Nephrol. 2020;21(1):130.

152. Nahar P, Kannuri NK, Mikkilineni S, Murthy GVS, Phillimore P. mHealth and the management of chronic conditions in rural areas: a note of caution from southern India. Anthropology & Medicine. 2017;24(1):1-16.

153. Nair PP, Aghoram R, Thomas B, Bharadwaj B, Chinnakali P. Video teleconsultation services for persons with epilepsy during COVID-19 pandemic: An exploratory study from public tertiary care hospital in Southern India on feasibility, satisfaction, and effectiveness. Epilepsy Behav. 2021;117:107863.

154. Najm A, Lempp H, Gossec L, Berenbaum F, Nikiphorou E. Needs, Experiences, and Views of People With Rheumatic and Musculoskeletal Diseases on Self-Management Mobile Health Apps: Mixed Methods Study. JMIR Mhealth Uhealth. 2020;8(4):e14351.

155. Nancarrow S, Banbury A, Buckley J. Evaluation of a National Broadband Network-enabled Telehealth trial for older people with chronic disease. Australian Health Review. 2016;40(6):641-8.

156. Ng G, Tan N, Bahadin J, Shum E, Tan SW. Development of an Automated Healthcare Kiosk for the Management of Chronic Disease Patients in the Primary Care Setting. J Med Syst. 2016;40(7):169.

157. Niendam TA, Tully LM, Iosif AM, Kumar D, Nye KE, Denton JC, et al. Enhancing early psychosis treatment using smartphone technology: A longitudinal feasibility and validity study. J Psychiatr Res. 2018;96:239-46.

158. Nohra RG, Chaaban T, Sacre H, Salameh P, Aoun Bacha Z, Le Bon Chami B, et al. Evaluating the Feasibility and Pretesting the Impact of an Educational and Telemonitoring Program for COPD Patients in Lebanon. Int J Chron Obstruct Pulmon Dis. 2022;17:949-65.

159. Nordin C, Michaelson P, Eriksson MK, Gard G. It's about me: Patients' experiences of patient participation in the web behavior change program for activity in combination with multimodal pain rehabilitation. Journal of Medical Internet Research. 2017;19(1):e22.

160. Nyberg A, Tistad M, Wadell K. Can the COPD web be used to promote self-management in patients with COPD in swedish primary care: a controlled pragmatic pilot trial with 3 month- and 12 month follow-up. Scand J Prim Health Care. 2019;37(1):69-82.

161. O'Connor SR, Treanor C, Ward E, Wickens RA, O'Connell A, Culliford LA, et al. Patient Acceptability of Home Monitoring for Neovascular Age-Related Macular Degeneration Reactivation: A Qualitative Study. Int J Environ Res Public Health. 2022;19(20).

162. Ong SW, Jassal SV, Miller JA, Porter EC, Cafazzo JA, Seto E, et al. Integrating a Smartphone-Based Self-Management System into Usual Care of Advanced CKD. Clin J Am Soc Nephrol. 2016;11(6):1054-62.

163. Ong SW, Kaushal A, Pariser P, Chan CT. An Integrated Kidney Care eConsult Practice Model: Results from the iKinect Project. Am J Nephrol. 2019;50(4):262-71.

164. Or C, Tao D. A 3-Month Randomized Controlled Pilot Trial of a Patient-Centered, Computer-Based Self-Monitoring System for the Care of Type 2 Diabetes Mellitus and Hypertension. Journal of Medical Systems. 2016;40(4):1-13.

165. Paldán K, Steinmetz M, Simanovski J, Rammos C, Ullrich G, Jánosi RA, et al. Supervised Exercise Therapy Using Mobile Health Technology in Patients With Peripheral Arterial Disease: Pilot Randomized Controlled Trial. JMIR Mhealth Uhealth. 2021;9(8):e24214.

166. Palermo TM, de la Vega R, Murray C, Law E, Zhou C. A digital health psychological intervention (WebMap Mobile) for children and adolescents with chronic pain: Results of a hybrid effectiveness-implementation stepped-wedge cluster randomized trial. Pain. 2020;161(12):2763-74.

167. Panagopoulos C, Malli F, Menychtas A, Smyrli EP, Georgountzou A, Daniil Z, et al. Utilizing a Homecare Platform for Remote Monitoring of Patients with Idiopathic Pulmonary Fibrosis. Adv Exp Med Biol. 2017;989:177-87.

168. Pariser P, Pham TT, Brown JB, Stewart M, Charles J. Connecting People With Multimorbidity to Interprofessional Teams Using Telemedicine. Ann Fam Med. 2019;17(Suppl 1):S57-s62.

169. Park SK, Bang CH, Lee SH. Evaluating the effect of a smartphone app-based self-management program for people with COPD: A randomized controlled trial. Appl Nurs Res. 2020;52:151231.

170. Passardi A, Foca F, Caffo O, Tondini CA, Zambelli A, Vespignani R, et al. A Remote Monitoring System to Optimize the Home Management of Oral Anticancer Therapies (ONCO-TreC): Prospective Training–Validation Trial. J Med Internet Res. 2022;24(1):e27349.

171. Patzer RE, McPherson L, Basu M, Mohan S, Wolf M, Chiles M, et al. Effect of the iChoose Kidney decision aid in improving knowledge about treatment options among transplant candidates: A randomized controlled trial. Am J Transplant. 2018;18(8):1954-65.

172. Peiris D, Praveen D, Mogulluru K, Ameer MA, Raghu A, Li Q, et al. SMARThealth India: A stepped-wedge, cluster randomised controlled trial of a community health worker managed mobile health intervention for people assessed at high cardiovascular disease risk in rural India. PLoS One. 2019;14(3):e0213708.

173. Peters D, Davis S, Calvo RA, Sawyer SM, Smith L, Foster JM. Young People's Preferences for an Asthma Self-Management App Highlight Psychological Needs: A Participatory Study. J Med Internet Res. 2017;19(4):e113.

174. Peterson S. Telerehabilitation booster sessions and remote patient monitoring in the management of chronic low back pain: A case series. Physiother Theory Pract. 2018;34(5):393-402.

175. Picton P, Wiljer D, Urowitz S, Cafazzo JA. Engaging Patients in Online Self-Care Technologies for Chronic Disease Management. Healthc Q. 2016;18(4):55-61.

176. Piotrowicz E, Pencina MJ, Opolski G, Zareba W, Banach M, Kowalik I, et al. Effects of a 9-Week Hybrid Comprehensive Telerehabilitation Program on Long-term Outcomes in Patients With Heart Failure: The Telerehabilitation in Heart Failure Patients (TELEREH-HF) Randomized Clinical Trial. JAMA Cardiol. 2020;5(3):300-8.

177. Pludwinski S, Ahmad F, Wayne N, Ritvo P. Participant experiences in a smartphone-based health coaching intervention for type 2 diabetes: A qualitative inquiry. J Telemed Telecare. 2016;22(3):172-8.

178. Pratap A, Grant D, Vegesna A, Tummalacherla M, Cohan S, Deshpande C, et al. Evaluating the Utility of Smartphone-Based Sensor Assessments in Persons With Multiple Sclerosis in the Real-World Using an App (elevateMS): Observational, Prospective Pilot Digital Health Study. JMIR Mhealth Uhealth. 2020;8(10):e22108.

179. Prevodnik K, Hvalič-Touzery S, Dolničar V, Zaletel J, Laznik J, Petrovčič A. Experience of patients with chronic conditions with telemedicine in primary care: A focus group analysis. Obzornik Zdravstvene Nege. 2022;56(4):246-63.

180. Puig J, Echeverría P, Lluch T, Herms J, Estany C, Bonjoch A, et al. A Specific Mobile Health Application for Older HIV-Infected Patients: Usability and Patient's Satisfaction. Telemed J E Health. 2021;27(4):432-40.

181. Quaedackers L, De Wit J, Pillen S, Van Gilst M, Batalas N, Lammers GJ, et al. A Mobile App for Longterm Monitoring of Narcolepsy Symptoms: Design, Development, and Evaluation. JMIR Mhealth Uhealth. 2020;8(1):e14939.

182. Racioppi A, Dalton T, Ramalingam S, Romero K, Ren Y, Bohannon L, et al. Assessing the Feasibility of a Novel mHealth App in Hematopoietic Stem Cell Transplant Patients. Transplant Cell Ther. 2021;27(2):181.e1-.e9.

183. Raeside R, Singleton AC, Todd A, Partridge SR, Hyun KK, Kulas H, et al. Lung Support Service: Implementation of a Nationwide Text Message Support Program for People with Chronic Respiratory Disease during the COVID-19 Pandemic. Int J Environ Res Public Health. 2022;19(24).

184. Raghu A, Praveen D, Peiris D, Tarassenko L, Clifford G. Engineering a mobile health tool for resource-poor settings to assess and manage cardiovascular disease risk: SMARThealth study. BMC Med Inform Decis Mak. 2015;15:36.

185. Reading Turchioe M, Grossman LV, Baik D, Lee CS, Maurer MS, Goyal P, et al. Older adults can successfully monitor symptoms using an inclusively designed mobile application. Journal of the American Geriatrics Society. 2020;68(6):1313-8.

186. Roca S, Lozano ML, García J, Alesanco Á. Validation of a Virtual Assistant for Improving Medication Adherence in Patients with Comorbid Type 2 Diabetes Mellitus and Depressive Disorder. Int J Environ Res Public Health. 2021;18(22).

187. Rodriguez Hermosa JL, Fuster Gomila A, Puente Maestu L, Amado Diago CA, Callejas González FJ, Malo De Molina Ruiz R, et al. Compliance and Utility of a Smartphone App for the Detection of Exacerbations in Patients With Chronic Obstructive Pulmonary Disease: Cohort Study. JMIR Mhealth Uhealth. 2020;8(3):e15699.

188. Rollo ME, Ash S, Lyons-Wall P, Russell AW. Evaluation of a Mobile Phone Image-Based Dietary Assessment Method in Adults with Type 2 Diabetes. Nutrients. 2015;7(6):4897-910.

189. Rothgangel A, Braun S, Smeets R, Beurskens A. Feasibility of a traditional and teletreatment approach to mirror therapy in patients with phantom limb pain: a process evaluation performed alongside a randomized controlled trial. Clin Rehabil. 2019;33(10):1649-60.

190. Rudin RS, Fanta CH, Qureshi N, Duffy E, Edelen MO, Dalal AK, et al. A Clinically Integrated mHealth App and Practice Model for Collecting Patient-Reported Outcomes between Visits for Asthma Patients: Implementation and Feasibility. Appl Clin Inform. 2019;10(5):783-93.

191. Rudolf I, Pieper K, Nolte H, Junge S, Dopfer C, Sauer-Heilborn A, et al. Assessment of a Mobile App by Adolescents and Young Adults With Cystic Fibrosis: Pilot Evaluation. JMIR Mhealth Uhealth. 2019;7(11):e12442.

192. Santo K, Singleton A, Rogers K, Thiagalingam A, Chalmers J, Chow CK, et al. Medication reminder applications to improve adherence in coronary heart disease: a randomised clinical trial. Heart. 2019;105(4):323-9.

193. Schneider T, Panzera AD, Martinasek M, McDermott R, Couluris M, Lindenberger J, et al. Physicians' perceptions of mobile technology for enhancing asthma care for youth. J Child Health Care. 2016;20(2):153-63.

194. Schougaard LM, Larsen LP, Jessen A, Sidenius P, Dorflinger L, de Thurah A, et al. AmbuFlex: tele-patient-reported outcomes (telePRO) as the basis for follow-up in chronic and malignant diseases. Qual Life Res. 2016;25(3):525-34.

195. Scriven H, Doherty DP, Ward EC. Evaluation of a multisite telehealth group model for persistent pain management for rural/remote participants. Rural Remote Health. 2019;19(1):4710.

196. Selman L, McDermott K, Donesky D, Citron T, Howie-Esquivel J. Appropriateness and acceptability of a Tele-Yoga intervention for people with heart failure and chronic obstructive pulmonary disease: qualitative findings from a controlled pilot study. BMC Complement Altern Med. 2015;15:21.

197. Seneviratne MG, Hersch F, Peiris DP. HealthNavigator: a mobile application for chronic disease screening and linkage to services at an urban Primary Health Network. Aust J Prim Health. 2018;24(2):116-22.

198. Seo NJ, Enders LR, Fortune A, Cain S, Vatinno AA, Schuster E, et al. Phase I Safety Trial: Extended Daily Peripheral Sensory Stimulation Using a Wrist-Worn Vibrator in Stroke Survivors. Transl Stroke Res. 2020;11(2):204-13.

199. Sherwin LB, Deroche CB, Yevu-Johnson J, Matteson-Kome M, Bechtold M, Jahnke I, et al. Usability Evaluation of a Smartphone Medication Reminder Application in Patients Treated With Short-term Antibiotic. CIN: Computers, Informatics, Nursing. 2021;39(10):547-53.

200. Shin S, Yeom CW, Shin C, Shin JH, Jeong JH, Shin JU, et al. Activity monitoring using a mHealth device and correlations with psychopathology in patients with chronic schizophrenia. Psychiatry Res. 2016;246:712-8.

201. Singh G, Nimmon L, Sawatzky B, Ben Mortenson W. Barriers and Facilitators to eHealth Technology Use Among Community-Dwelling Individuals With Spinal Cord Injury: A Qualitative Study. Top Spinal Cord Inj Rehabil. 2022;28(2):196-204.

202. Sjöström M, Umefjord G, Stenlund H, Carlbring P, Andersson G, Samuelsson E. Internet-based treatment of stress urinary incontinence: 1- and 2-year results of a randomized controlled trial with a focus on pelvic floor muscle training. BJU Int. 2015;116(6):955-64.

203. Slevin P, Kessie T, Cullen J, Butler MW, Donnelly SC, Caulfield B. Exploring the barriers and facilitators for the use of digital health technologies for the management of COPD: a qualitative study of clinician perceptions. Qjm. 2020;113(3):163-72.

204. Sloots J, Bakker M, van der Palen J, Eijsvogel M, van der Valk P, Linssen G, et al. Adherence to an eHealth Self-Management Intervention for Patients with Both COPD and Heart Failure: Results of a Pilot Study. Int J Chron Obstruct Pulmon Dis. 2021;16:2089-103.

205. Smithson R, Roche E, Wicker C. Virtual models of chronic disease management: lessons from the experiences of virtual care during the COVID-19 response. Australian Health Review. 2021;45(3):311-6.

206. Sobrinho A, da Silva LD, Perkusich A, Pinheiro ME, Cunha P. Design and evaluation of a mobile application to assist the self-monitoring of the chronic kidney disease in developing countries. BMC Med Inform Decis Mak. 2018;18(1):7.

207. Son YJ, Oh S, Kim EY. Patients' needs and perspectives for using mobile phone interventions to improve heart failure self-care: A qualitative study. J Adv Nurs. 2020;76(9):2380-90.

208. Song CE, An M. The Self-management Smartphone Application for Cancer Survivors, ReLive: Development and Usability Testing. Comput Inform Nurs. 2021;39(6):312-20.

209. Song T, Liu F, Deng N, Qian S, Cui T, Guan Y, et al. A Comprehensive 6A Framework for Improving Patient Self-Management of Hypertension Using mHealth Services: Qualitative Thematic Analysis. J Med Internet Res. 2021;23(6):e25522.

210. Sönnerfors P, Wadell K, Dohrn IM, Nyberg A, Runold M, Halvarsson A. Use of an eHealth tool for exercise training and online contact in people with severe chronic obstructive pulmonary disease on long-term oxygen treatment: A feasibility study. Health Informatics J. 2020;26(4):3184-200.

211. Souza-Silva MVR, Domingues MLP, Chagas VS, Pereira DN, de Sá LC, Almeida MSS, et al. Implementation of a text messaging intervention to patients on warfarin therapy in Brazilian primary care units: a quasi-experimental study. BMC Prim Care. 2022;23(1):54.

212. Steare T, Giorgalli M, Free K, Harju-Seppänen J, Akther S, Eskinazi M, et al. A qualitative study of stakeholder views on the use of a digital app for supported self-management in early intervention services for psychosis. BMC Psychiatry. 2021;21(1):311.

213. Stonbraker S, Cho H, Hermosi G, Pichon A, Schnall R. Usability Testing of a mHealth App to Support Self-Management of HIV-Associated Non-AIDS Related Symptoms. Stud Health Technol Inform. 2018;250:106-10.

214. Stroupe KT, Martinez R, Hogan TP, Evans CT, Scholten J, Bidelspach D, et al. Health Care Utilization and Costs of Veterans Evaluated for Traumatic Brain Injury Through Telehealth. Telemed J E Health. 2019;25(12):1144-53.

215. Sulkowski M, Luetkemeyer AF, Wyles DL, Martorell C, Muir A, Weisberg I, et al. Impact of a digital medicine programme on hepatitis C treatment adherence and efficacy in adults at high risk for non-adherence. Aliment Pharmacol Ther. 2020;51(12):1384-96.

216. Sumino K, Locke ER, Magzamen S, Gylys-Colwell I, Humblet O, Nguyen HQ, et al. Use of a Remote Inhaler Monitoring Device to Measure Change in Inhaler Use with Chronic Obstructive Pulmonary Disease Exacerbations. J Aerosol Med Pulm Drug Deliv. 2018;31(3):191-8.

217. Talal AH, Jaanimägi U, Davis K, Bailey J, Bauer BM, Dharia A, et al. Facilitating engagement of persons with opioid use disorder in treatment for hepatitis C virus infection via telemedicine: Stories of onsite case managers. J Subst Abuse Treat. 2021;127:108421.

218. Talal AH, Markatou M, Sofikitou EM, Brown LS, Perumalswami P, Dinani A, et al. Patient-centered HCV care via telemedicine for individuals on medication for opioid use disorder: Telemedicine for Evaluation, Adherence and Medication for Hepatitis C (TEAM-C). Contemp Clin Trials. 2022;112:106632.

219. Talboom-Kamp E, Holstege MS, Chavannes NH, Kasteleyn MJ. Effects of use of an eHealth platform e-Vita for COPD patients on disease specific quality of life domains. Respir Res. 2019;20(1):146.

220. Tassorelli C, Jensen R, Allena M, De Icco R, Katsarava Z, Miguel Lainez J, et al. The added value of an electronic monitoring and alerting system in the management of medication-overuse headache: A controlled multicentre study. Cephalalgia. 2017;37(12):1115-25.

221. Taylor AH, Taylor RS, Ingram WM, Anokye N, Dean S, Jolly K, et al. Adding web-based behavioural support to exercise referral schemes for inactive adults with chronic health conditions: the e-coachER RCT. Health Technol Assess. 2020;24(63):1-106.

222. Taylor S, Allsop MJ, Bekker HL, Bennett MI, Bewick BM. Identifying professionals' needs in integrating electronic pain monitoring in community palliative care services: An interview study. Palliat Med. 2017;31(7):661-70.

223. Thomas RM, Locke ER, Woo DM, Nguyen EHK, Press VG, Layouni TA, et al. Inhaler Training Delivered by Internet-Based Home Videoconferencing Improves Technique and Quality of Life. Respir Care. 2017;62(11):1412-22.

224. Tillis W, Bond WF, Svendsen J, Guither S. Implementation of Activity Sensor Equipment in the Homes of Chronic Obstructive Pulmonary Disease Patients. Telemed J E Health. 2017;23(11):920-9.

225. Tran C, Dicker A, Leiby B, Gressen E, Williams N, Jim H. Utilizing Digital Health to Collect Electronic Patient-Reported Outcomes in Prostate Cancer: Single-Arm Pilot Trial. J Med Internet Res. 2020;22(3):e12689.

226. Trosini-Désert V, Lafoeste H, Regard L, Malrin R, Galarza-Jimenez MA, Amarilla CE, et al. A Telemedicine Intervention to Ensure the Correct Usage of Inhaler Devices. Telemed J E Health. 2020;26(11):1336-44.

227. Tucker S, Abbott L, Anderson R, Eppen K, Laroche H, Paelmo E, et al. Implementing Follow-Along Physical Activity Videos with People Living With Chronic Conditions: A Feasibility Study. Worldviews Evid Based Nurs. 2019;16(5):352-61.

228. Turino C, Benítez ID, Rafael-Palou X, Mayoral A, Lopera A, Pascual L, et al. Management and Treatment of Patients With Obstructive Sleep Apnea Using an Intelligent Monitoring System Based on Machine Learning Aiming to Improve Continuous Positive Airway Pressure Treatment Compliance: Randomized Controlled Trial. J Med Internet Res. 2021;23(10):e24072.

229. Tyrrell JS, Redshaw CH. Physical Activity in Ankylosing Spondylitis: evaluation and analysis of an eHealth tool. J Innov Health Inform. 2016;23(2):169.

230. Urech C, Grossert A, Alder J, Scherer S, Handschin B, Kasenda B, et al. Web-Based Stress Management for Newly Diagnosed Patients With Cancer (STREAM): A Randomized, Wait-List Controlled Intervention Study. Journal of Clinical Oncology. 2018;36(8):780-8.

231. van den Wijngaart LS, Geense WW, Boehmer AL, Brouwer ML, Hugen CA, van Ewijk BE, et al. Barriers and Facilitators When Implementing Web-Based Disease Monitoring and Management as a Substitution for Regular Outpatient Care in Pediatric Asthma: Qualitative Survey Study. J Med Internet Res. 2018;20(10):e284.

232. Vasi S, Advocat J, Adaji A, Russell G. Building quality chronic illness care: implementation of a web-based care plan. Australian Journal of Primary Health. 2020;26(2):173-7.

233. Vatnøy TK, Thygesen E, Dale B. Telemedicine to support coping resources in home-living patients diagnosed with chronic obstructive pulmonary disease: Patients' experiences. J Telemed Telecare. 2017;23(1):126-32.

234. Verwey R, van der Weegen S, Spreeuwenberg M, Tange H, van der Weijden T, de Witte L. Process evaluation of physical activity counselling with and without the use of mobile technology: A mixed methods study. Int J Nurs Stud. 2016;53:3-16.

235. Vest BM, Hall VM, Kahn LS, Heider AR, Maloney N, Singh R. Nurse perspectives on the implementation of routine telemonitoring for high-risk diabetes patients in a primary care setting. Prim Health Care Res Dev. 2017;18(1):3-13.

236. Vijayasundaram S, Karthikeyan P, Mehta SD. Proficiency of virtual follow-up amongst tinnitus patients who underwent intratympanic steroid therapy amidst COVID 19 pandemic. Am J Otolaryngol. 2020;41(6):102680.

237. Wakefield BJ, Alexander G, Dohrmann M, Richardson J. Design and Evaluation of a Web-Based Symptom Monitoring Tool for Heart Failure. Comput Inform Nurs. 2017;35(5):248-54.

238. Wali S, Guessi Margarido M, Shah A, Ware P, McDonald M, O'Sullivan M, et al. Expanding Telemonitoring in a Virtual World: A Case Study of the Expansion of a Heart Failure Telemonitoring Program During the COVID-19 Pandemic. J Med Internet Res. 2021;23(1):e26165.

239. Wallace T, Morris JT, Glickstein R, Anderson RK, Gore RK. Implementation of a Mobile Technology-Supported Diaphragmatic Breathing Intervention in Military mTBI With PTSD. J Head Trauma Rehabil. 2022;37(3):152-61.

240. Walsh DM, Moran K, Cornelissen V, Buys R, Cornelis N, Woods C. Electronic Health Physical Activity Behavior Change Intervention to Self-Manage Cardiovascular Disease: Qualitative Exploration of Patient and Health Professional Requirements. J Med Internet Res. 2018;20(5):e163.

241. Wannheden C, Stenfors T, Stenling A, von Thiele Schwarz U. Satisfied or Frustrated? A Qualitative Analysis of Need Satisfying and Need Frustrating Experiences of Engaging With Digital Health Technology in Chronic Care. Front Public Health. 2020;8:623773.

242. Ware P, Shah A, Ross HJ, Logan AG, Segal P, Cafazzo JA, et al. Challenges of Telemonitoring Programs for Complex Chronic Conditions: Randomized Controlled Trial With an Embedded Qualitative Study. J Med Internet Res. 2022;24(1):e31754.

243. Wei KS, Ibrahim NE, Kumar AA, Jena S, Chew V, Depa M, et al. Habits Heart App for Patient Engagement in Heart Failure Management: Pilot Feasibility Randomized Trial. JMIR Mhealth Uhealth. 2021;9(1):e19465.

244. Welzel FD, Bär J, Stein J, Löbner M, Pabst A, Luppa M, et al. Using a brief web-based 5A intervention to improve weight management in primary care: results of a cluster-randomized controlled trial. BMC Fam Pract. 2021;22(1):61.

245. Wheeler TS, Michael Vallis T, Giacomantonio NB, Abidi SR. Feasibility and usability of an ontology-based mobile intervention for patients with hypertension. International Journal of Medical Informatics. 2018;119:8-16.

246. Wickerson L, Helm D, Gottesman C, Rozenberg D, Singer LG, Keshavjee S, et al. Telerehabilitation for Lung Transplant Candidates and Recipients During the COVID-19 Pandemic: Program Evaluation. JMIR Mhealth Uhealth. 2021;9(6):e28708.

247. Wright S, Thompson N, Yadrich D, Bruce A, Bonar JRM, Spaulding R, et al. Using telehealth to assess depression and suicide ideation and provide mental health interventions to groups of chronically ill adolescents and young adults. Research in Nursing & Health. 2021;44(1):129-37.

248. Yeh CH, Kawi J, Ni A, Christo P. Evaluating Auricular Point Acupressure for Chronic Low Back Pain Self-Management Using Technology: A Feasibility Study. Pain Manag Nurs. 2022;23(3):301-10.

249. Zahid M, Gallant NL, Hadjistavropoulos T, Stroulia E. Behavioral Pain Assessment Implementation in Long-Term Care Using a Tablet App: Case Series and Quasi-Experimental Design. JMIR Mhealth Uhealth. 2020;8(4):e17108.

250. Zand A, Nguyen A, Reynolds C, Khandadash A, Esrailian E, Hommes D. Patient Experience and Satisfaction with an e-Health Care Management Application for Inflammatory Bowel Diseases. Int J Environ Res Public Health. 2021;18(22).

251. Zetterqvist V, Gentili C, Rickardsson J, Sorensen I, Wicksell RK. Internet-delivered acceptance and commitment therapy for adolescents with chronic pain and their parents: A nonrandomized pilot trial. Journal of Pediatric Psychology. 2020;45(9):990-1004.

252. Zhang J, Mihai C, Tüshaus L, Scebba G, Distler O, Karlen W. Wound Image Quality From a Mobile Health Tool for Home-Based Chronic Wound Management With Real-Time Quality Feedback: Randomized Feasibility Study. JMIR Mhealth Uhealth. 2021;9(7):e26149.

# S5. Type of study


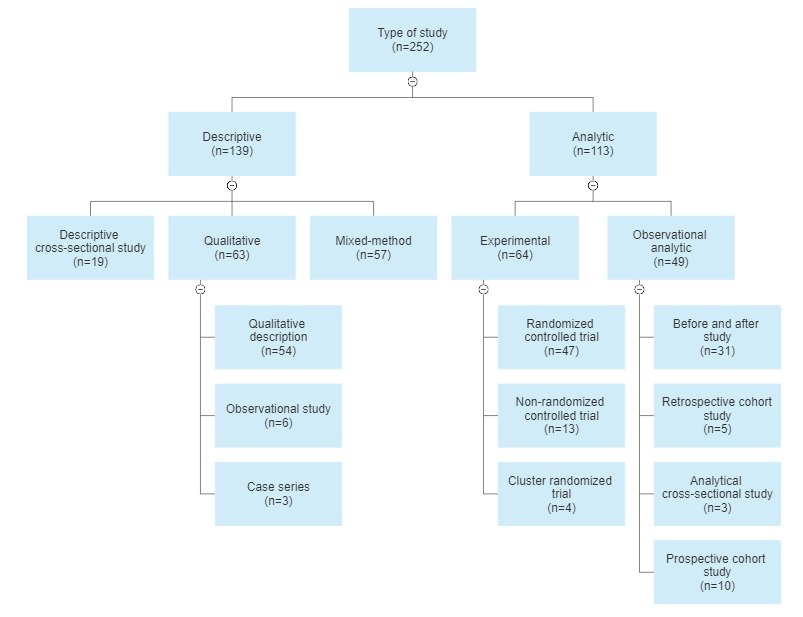


# S6. Outcome measures used to evaluate implementation success and effectiveness of the digital health innovation

Implementation outcomes indicate the success (or otherwise) of implementing or embedding the digital health innovation. The eight implementation outcomes can be grouped into (a) outcomes from an end-user perspective that are a function of innovation design (acceptability, adoption, appropriateness, feasibility), (b) implementation process (fidelity), and (c) outcomes that foreshadow embedment in routine practice from an organizational perspective (cost and cost-effectiveness, penetration, sustainability).

Service outcomes and patient outcomes indicate the effectiveness of the digital health innovation in impacting service delivery/patient care and patient health/well-being, respectively. Examples of these outcomes include: patient safety indicators, quantifiable health outcomes, patient satisfaction, health-related quality of life, patient empowerment, and patient knowledge.

The outcome level listed in the table below was determined by study authors’ descriptions of their findings. For example, acceptability was rated as “high” if study authors indicated that more than half of target users found the innovation acceptable; “mixed” if it was inconclusive whether majority of target users found it acceptable (e.g. most patients found the innovation acceptable, but most healthcare workers did not); and “low” if less than half of target users found it acceptable.

Quality of life was rated “high” if study authors reported a significant improvement in target users’ quality of life post implementation; “mixed” if the improvement was not significant; and “low” if there was a drop in target users’ quality of life, and so forth.

| **Outcomes in implementation research** | | | |
| --- | --- | --- | --- |
| **Type of innovation^a^** | **Outcome** | **Outcome level** | **Number of papers** |
| eHealth (n=61) | Implementation outcomes | | |
|  | Acceptability | High | 38 |
|  |  | Mixed | 0 |
|  |  | Low | 1 |
|  |  | Not investigated | 22 |
|  | Adoption | High | 10 |
|  |  | Mixed | 0 |
|  |  | Low | 0 |
|  |  | Not investigated | 51 |
|  | Appropriateness | High | 0 |
|  |  | Mixed | 0 |
|  |  | Low | 0 |
|  |  | Not investigated | 61 |
|  | Feasibility | High | 7 |
|  |  | Mixed | 1 |
|  |  | Low | 2 |
|  |  | Not investigated | 51 |
|  | Fidelity | High | 1 |
|  |  | Mixed | 0 |
|  |  | Low | 0 |
|  |  | Not investigated | 60 |
|  | Cost^b^ | High | 0 |
|  |  | Mixed | 0 |
|  |  | Low | 1 |
|  |  | Not investigated | 60 |
|  | Penetration | High | 0 |
|  |  | Mixed | 0 |
|  |  | Low | 0 |
|  |  | Not investigated | 61 |
|  | Sustainability | High | 1 |
|  |  | Mixed | 0 |
|  |  | Low | 0 |
|  |  | Not investigated | 60 |
|  | Patient outcomes | | |
|  | Health outcomes | High | 15 |
|  |  | Mixed | 8 |
|  |  | Low | 1 |
|  |  | Not investigated | 37 |
|  | Satisfaction | High | 6 |
|  |  | Mixed | 0 |
|  |  | Low | 0 |
|  |  | Not investigated | 55 |
|  | Quality of life | High | 4 |
|  |  | Mixed | 3 |
|  |  | Low | 0 |
|  |  | Not investigated | 54 |
|  | Patient empowerment | High | 1 |
|  |  | Mixed | 0 |
|  |  | Low | 0 |
|  |  | Not investigated | 60 |
|  | Patient knowledge | High | 2 |
|  |  | Mixed | 0 |
|  |  | Low | 0 |
|  |  | Not investigated | 59 |
|  | Service outcomes | | |
|  | Safety | High | 1 |
|  |  | Mixed | 0 |
|  |  | Low | 0 |
|  |  | Not investigated | 60 |
| mHealth (n=107) | Implementation outcomes | | |
|  | Acceptability | High | 70 |
|  |  | Mixed | 4 |
|  |  | Low | 0 |
|  |  | Not investigated | 33 |
|  | Adoption | High | 14 |
|  |  | Mixed | 0 |
|  |  | Low | 1 |
|  |  | Not investigated | 92 |
|  | Appropriateness | High | 0 |
|  |  | Mixed | 0 |
|  |  | Low | 2 |
|  |  | Not investigated | 105 |
|  | Feasibility | High | 16 |
|  |  | Mixed | 0 |
|  |  | Low | 2 |
|  |  | Not investigated | 89 |
|  | Fidelity | High | 0 |
|  |  | Mixed | 0 |
|  |  | Low | 0 |
|  |  | Not investigated | 107 |
|  | Cost^b^ | High | 0 |
|  |  | Mixed | 0 |
|  |  | Low | 2 |
|  |  | Not investigated | 105 |
|  | Penetration | High | 0 |
|  |  | Mixed | 0 |
|  |  | Low | 0 |
|  |  | Not investigated | 107 |
|  | Sustainability | High | 0 |
|  |  | Mixed | 0 |
|  |  | Low | 0 |
|  |  | Not investigated | 107 |
|  | Patient outcomes | | |
|  | Health outcomes | High | 25 |
|  |  | Mixed | 8 |
|  |  | Low | 2 |
|  |  | Not investigated | 72 |
|  | Satisfaction | High | 15 |
|  |  | Mixed | 0 |
|  |  | Low | 0 |
|  |  | Not investigated | 92 |
|  | Quality of life | High | 5 |
|  |  | Mixed | 4 |
|  |  | Low | 0 |
|  |  | Not investigated | 98 |
|  | Patient empowerment | High | 2 |
|  |  | Mixed | 0 |
|  |  | Low | 0 |
|  |  | Not investigated | 105 |
|  | Patient knowledge | High | 1 |
|  |  | Mixed | 0 |
|  |  | Low | 0 |
|  |  | Not investigated | 106 |
|  | Service outcomes | | |
|  | Safety | High | 2 |
|  |  | Mixed | 0 |
|  |  | Low | 0 |
|  |  | Not investigated | 105 |
| Telehealth/Telemedicine (n=97) | Implementation outcomes | | |
|  | Acceptability | High | 59 |
|  |  | Mixed | 0 |
|  |  | Low | 2 |
|  |  | Not investigated | 36 |
|  | Adoption | High | 4 |
|  |  | Mixed | 1 |
|  |  | Low | 0 |
|  |  | Not investigated | 92 |
|  | Appropriateness | High | 2 |
|  |  | Mixed | 0 |
|  |  | Low | 0 |
|  |  | Not investigated | 95 |
|  | Feasibility | High | 13 |
|  |  | Mixed | 0 |
|  |  | Low | 1 |
|  |  | Not investigated | 83 |
|  | Fidelity | High | 3 |
|  |  | Mixed | 0 |
|  |  | Low | 0 |
|  |  | Not investigated | 94 |
|  | Cost^b^ | High | 0 |
|  |  | Mixed | 1 |
|  |  | Low | 4 |
|  |  | Not investigated | 92 |
|  | Penetration | High | 0 |
|  |  | Mixed | 1 |
|  |  | Low | 0 |
|  |  | Not investigated | 96 |
|  | Sustainability | High | 0 |
|  |  | Mixed | 1 |
|  |  | Low | 1 |
|  |  | Not investigated | 95 |
|  | Patient outcomes | | |
|  | Health outcomes | High | 29 |
|  |  | Mixed | 7 |
|  |  | Low | 0 |
|  |  | Not investigated | 61 |
|  | Satisfaction | High | 22 |
|  |  | Mixed | 0 |
|  |  | Low | 1 |
|  |  | Not investigated | 74 |
|  | Quality of life | High | 4 |
|  |  | Mixed | 5 |
|  |  | Low | 1 |
|  |  | Not investigated | 87 |
|  | Patient empowerment | High | 0 |
|  |  | Mixed | 0 |
|  |  | Low | 0 |
|  |  | Not investigated | 97 |
|  | Patient knowledge | High | 0 |
|  |  | Mixed | 0 |
|  |  | Low | 0 |
|  |  | Not investigated | 97 |
|  | Service outcomes | | |
|  | Safety | High | 3 |
|  |  | Mixed | 0 |
|  |  | Low | 0 |
|  |  | Not investigated | 94 |
| Wearables (n=17) | Implementation outcomes | | |
|  | Acceptability | High | 7 |
|  |  | Mixed | 1 |
|  |  | Low | 0 |
|  |  | Not investigated | 9 |
|  | Adoption | High | 2 |
|  |  | Mixed | 0 |
|  |  | Low | 0 |
|  |  | Not investigated | 15 |
|  | Appropriateness | High | 0 |
|  |  | Mixed | 0 |
|  |  | Low | 0 |
|  |  | Not investigated | 0 |
|  | Feasibility | High | 1 |
|  |  | Mixed | 0 |
|  |  | Low | 0 |
|  |  | Not investigated | 16 |
|  | Fidelity | High | 0 |
|  |  | Mixed | 0 |
|  |  | Low | 0 |
|  |  | Not investigated | 17 |
|  | Cost^b^ | High | 0 |
|  |  | Mixed | 0 |
|  |  | Low | 0 |
|  |  | Not investigated | 17 |
|  | Penetration | High | 0 |
|  |  | Mixed | 0 |
|  |  | Low | 0 |
|  |  | Not investigated | 17 |
|  | Sustainability | High | 0 |
|  |  | Mixed | 0 |
|  |  | Low | 0 |
|  |  | Not investigated | 17 |
|  | Patient outcomes | | |
|  | Health outcomes | High | 2 |
|  |  | Mixed | 1 |
|  |  | Low | 0 |
|  |  | Not investigated | 14 |
|  | Satisfaction | High | 1 |
|  |  | Mixed | 0 |
|  |  | Low | 0 |
|  |  | Not investigated | 16 |
|  | Quality of life | High | 1 |
|  |  | Mixed | 0 |
|  |  | Low | 0 |
|  |  | Not investigated | 16 |
|  | Patient empowerment | High | 0 |
|  |  | Mixed | 0 |
|  |  | Low | 0 |
|  |  | Not investigated | 17 |
|  | Patient knowledge | High | 0 |
|  |  | Mixed | 0 |
|  |  | Low | 0 |
|  |  | Not investigated | 17 |
|  | Service outcomes | | |
|  | Safety | High | 2 |
|  |  | Mixed | 0 |
|  |  | Low | 0 |
|  |  | Not investigated | 15 |
| ^a^Some studies discussed more than one innovation.  ^b^Only Raeside 2022 reported implementation cost; other studies reported cost of innovation. | | | |
|  |  |  |  |
